# Double Role of Diphenylpyridine Derivatives as Fluorescent Sensors for Monitoring Photopolymerization and the Determination of the Efficiencies of the Generation of Superacids by Cationic Photoinitiators

**DOI:** 10.3390/s20113043

**Published:** 2020-05-27

**Authors:** Monika Topa, Filip Petko, Mariusz Galek, Joanna Ortyl

**Affiliations:** 1 Faculty of Chemical Engineering and Technology, Cracow University of Technology, Warszawska 24, 30-155 Cracow, Poland; monika.topa@doktorant.pk.edu.pl; 2 Photo HiTech Ltd., Bobrzyńskiego 14, 30-348 Cracow, Poland; filip.petko@photohitech.com (F.P.); mariusz.galek@photohitech.com (M.G.)

**Keywords:** fluorescent molecular sensors, photopolymerization, fluorescence probe technology, cationic photopolymerization, free-radical photopolymerization, superacid generation, pH sensors

## Abstract

Novel fluorescent sensors with electron-donating or electron-withdrawing substituents incorporated into a chromophore group based on 2,6-diphenylpyridine were designed and synthesised. The spectroscopic properties of these compounds were studied. Moreover, the positive solvatochromism of 2,6-bis-(4-methylsulphanylphenyl)pyridine (PT-SCH_3_) in selected solvents was studied by measurement of the absorption and emission spectra and analysed using the Dimroth–Reichardt solvent parameter set. After that, the performance of a series of 2,6-diphenylpyridine derivatives as fluorescent molecular sensors for monitoring free-radical and cationic photopolymerization processes by the Fluorescence Probe Technique (FPT) was studied. As a consequence of this stage of research, the effect of substituents on the sensitivity of the 2,6-diphenylpyridine derivatives as sensors during photopolymerization has been evaluated and discussed. It has been found that compounds containing strong electron-donating substituent (PT-SCH_3_) slightly shift their fluorescence spectrum during the free-radical polymerization of monomer, which enables the monitoring of the polymerization progress using the fluorescence intensity ratio measured at two different wavelengths as the progress indicator. The position of the fluorescence spectrum of 2,6-diphenylpyridine derivatives with electron-withdrawing substituents is practically insensitive to changes occurring in their environment. Hence, it is recommended to use these compounds with different indicators of the progress of the photopolymerization process based on normalised intensity of fluorescence (I_max_/I_0_). Among the compounds studied, 2,6-bis(4-methylsulphanylphenyl)pyridine (PT-SCH_3_) turned out to be the best fluorescent sensor for the purpose of monitoring free-radical polymerization by FPT. Consequently, the dual application of the selected 2,6-diphenylpyridine derivatives is proposed: (a) as fluorescent sensors for monitoring the free-radical photopolymerization progress, and (b) as spectroscopic sensors for the determination of efficiencies of the generation of superacids by cationic photoinitiators during the cationic photopolymerization process. Finally, a new method for determining the relative efficiency of the photogeneration of superacids during the photo cleavage of onium salt has been devised and applied for the evaluation of the performance of 2,6-diphenylpyridine derivatives.

## 1. Introduction

Common fluorescent molecular sensors play an important role in practically every area of life. Fluorescent chemosensors have been around for about 150 years. Their pioneer is Goppelsröder, who, in 1867, reported the first fluorescent chemosensor for Al^3+^ [1]. In recent years, a lot of emphasis has been placed on the use of fluorescent sensors, especially in biology [2], biochemistry [3], biotechnology and medicine [4,5], including physiology, pharmacology and environmental sciences [6]. In particular, fluorescent chemosensors for biological and environmental cations [7], anions [8], small neutral molecules [9] and biomacromolecules [10], such as proteins and DNA [11], have been developed alongside rapid advancements in microscopic imaging technology [12]. For example, Schmuck et al. reported a pyrene-based peptide beacon—a fluorescent chemosensor that is intercalated with DNA [11]. The detection of an analyte using a fluorescent chemosensor is usually achieved by one or more common photophysical mechanisms, e.g., chelation-induced enhanced fluorescence (CHEF) [1], intramolecular charge transfer (ICT) [13], photoinduced electron transfer (PET) [14] and induced emission aggregation (AIE) [15].

In recent years, great emphasis has been placed on developing new fluorescent molecular sensors for detecting selected metal ions [3,16,17,18,19,20]. Liu et al. developed a novel 4-amino-1,8-naphthalimide-based fluorescent sensor with iminodiacetic acid as receptor, applied successfully to image Zn^2 +^ in living cells [3]. Song et al. developed a novel fluorescent sensor for Al^3+^ and Zn^2+^ based on a new europium complex with a 1,10-phenanthroline ligand [19]. The complex shows high sensitivity and selectivity towards Al^3+^ and Zn^2+^ in a methanol solution, which can be attributed to the Al^3+/^Zn^2+^ cation instead of Eu^3+^ in the complex [19]. In turn, Behnam Majidi et al. developed a dual-mode colorimetric-fluorescent sensor for the highly sensitive and selective detection of the Mg^2+^ ion in aqueous media [20].

Despite the progress made in this area, there are still several problems and challenges [21,22]. In order for these probes to be used in the above-mentioned fields of science, they should meet a number of requirements and respond appropriately to changes in the environment, i.e., changes in viscosity, polarity or pH [23,24]. What is more, if they are to be used in biological sciences, they should be non-toxic and also harmless to the human body. Therefore, new fluorescent sensors for biological applications are being intensively sought. Moreover, the popularity and development of technologies based on the use of fluorescent sensors go hand in hand with the development of spectrofluorometric techniques. This type of technique allows the study of even single sensor molecules and their interaction with other molecular entities under the influence of changes in various physicochemical parameters at the microscopic level. Thus, it distinguishes them from techniques that provide information at the macroscopic level. From a practical point of view, an additional and important feature of fluorescent techniques is the possibility of remote monitoring through the use of fibre optics and laser technologies. Due to the availability of spectroscopic techniques with high detection sensitivity and fast response time, it is possible to obtain accurate information about local physical, structural and chemical parameters of the reaction environment based on, among other things, the characteristics of the emission spectrum [25] (shape, width, intensity), quantum fluorescence performance [26] and sensor lifetimes [27]. In the field of polymer chemistry, techniques based on fluorescence spectroscopy and fluorescent probes are also successfully used. Such probes are compounds that are covalently unrelated to the polymer macromolecule and are able to provide information on the local physical, chemical and structural parameters of the microenvironment. Currently, due to the diversity and heterogeneous and dynamic nature of polymer systems, the big challenge is to study the processes taking place at the molecular and microscopic levels. While most polymeric materials are not fluorescent, these polymers can be tested by placing the appropriate fluorescent probe in them. This solution, in combination with fluorescent techniques (stationary, time-resolved, microscopy), provides non-invasive, non-destructive, in-situ and real-time studies of the structure and dynamics of the processes occurring in polymers. These are compounds that respond to changes in the environment. With regard to their application in the field of polymers [28], they are used in studies of the physicochemistry of polymers [8], as well as the kinetics of polymerization processes [26]. These probes can generally be grouped into several types, based on the following mechanisms: TICT (Twisted Intermolecular Charge Transfer) effect [29] (e.g., 4-dimethylamino benzonitrile and 4-dimethylaminobenzoic acid ethyl ester) [30]; sensors based on the ICT (Intermolecular Charge Transfer) effect [31,32], exciplex formation (e.g., pyrene); and changes in phosphorescence characteristics instead of fluorescence (i.e., phosphorescent probes). Fluorogenic probes are also known, which do not fluoresce in an unbound form, but strongly fluoresce after incorporation into the polymer structure (e.g., 1-phenyl-4-(4-cyano-1-naphthylmethylene) piperidine, commonly referred to as "Fluoroprobe") [33]. In addition, organic salt type probes (e.g., stilbazolium salts) [34] can also be distinguished, which show intermolecular excited electron transfer from the dimethylamino group to the pyridine or benzothiazole ring, but, unlike ICT or TICT probes, electron transfer does not cause a significant change in the dipole moment of excited states compared to the dipole moment these probes have in their ground state.

Currently, we have many methods for continuously monitoring the progress of photopolymerization. These allow monitoring of polymerization in real-time (on-line) upon irradiation to light. One of the famous and commonly used methods belonging to this group is isothermal differential scanning calorimetry (photo-DSC) [35,36,37]. Therefore, this method is unsuitable for determining the conversion of high-speed processes such as photopolymerization. Fourier-transform infrared spectroscopy in real-time (real-time-FT-IR) [38,39] is also a frequently used method, enabling the monitoring of the disappearance of absorbance bands at wavenumbers corresponding to the bands of a specific functional group. This method, due to its short response time, can be used to determine the conversion rates of photopolymerization. However, FT-IR also has limitations, such as sensitivity to light, humidity, and oxygen, which cause the results to be falsified. Therefore, fluorescence spectroscopy is gaining popularity.

The basis of this technique, called Fluorescence Probe Technology (FPT), is to measure changes in the probe’s emission spectrum as the environment changes [40]. During the photopolymerization process, due to the increase in system viscosity, there may be changes in the quantum efficiency of probe fluorescence, the location of the maximum intensity of the emission spectrum, fluorescence intensity and fluorescence polarization [41]. In turn, commonly used parameters to monitor the progress of the photopolymerization process in Fluorescence Probe Technology includes the so-called parameter of the ratio of fluorescence intensity (R) measured at two wavelengths located on both sides of the maximum fluorescence spectrum [42] and the parameter I_max_/I_0_ defined as the normalised fluorescence intensity measured at the maximum emission spectrum [43]. Parameter R illustrates the change in the shift of emission spectra during irradiation of the system. Typically, the ratio R is determined by dividing the intensity at a smaller wavelength (I_1_) by the intensity at a larger wavelength (I_2_). Fluorescence Probe Technology can be fruitfully deployed to investigate various processes and phenomena in polymer science, such as different types of polymerization processes [44,45,46,47,48], (including cross-linking and gel formation), irreversible film formation by physical interpenetration and the diffusion of polymer chains across interfaces, phase separation, and reversible transitions, such as glass transitions [43].

As part of this work, new 2,6-diphenylpyridines were developed as sensors for the determination of efficiencies of superacid generation by cationic photoinitiators using Fluorescence Probe Technology. Unlike the complicated structure of the pyridine terphenyls presented in a previous article [49], the pyridine terphenyls presented in this article are colourless in visible light, which is extremely important from an industrial point of view. Commercially available photoinitiators—iodonium salts, such as diphenyliodonium hexafluorophosphate (HIP)—and the commercially available monomer triethylene glycol divinyl ether (TEGDVE) were used for the cationic photopolymerization process. As a result of the cationic photopolymerization process involving photoinitiators, the emission spectrum was shifted towards longer wavelengths, which is directly related to the release of acid originating from the photoinitiator. The rate of acid evolution from the HIP photoinitiator was compared depending on the chemical structure of the sensors, i.e., from the nature of the substituent based on its electron-donating or electron-withdrawing character. Moreover, the impact of environmental pH changes and polarity on the emission spectra of selected 2,6-diphenylpyridine compounds was investigated. Finally, 2,6-diphenyl pyridine derivatives were also investigated as fluorescent sensors for monitoring the kinetics of the free-radical photopolymerization processes of the model trimethylpropane triacrylate monomer (TMPTA) as a process in which strong protic acid is not generated.

## 2. Materials and Methods

**Scheme 1:**
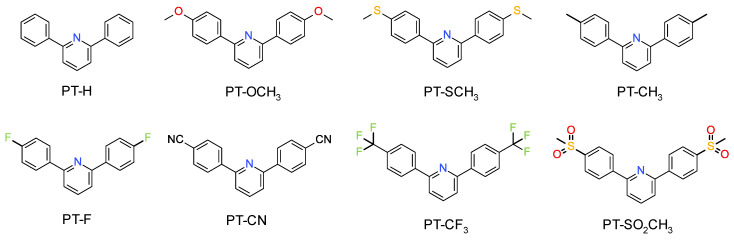
Structures of molecular fluorescent sensors.

Eight 2,6-diphenyl pyridine derivatives, differentiated in terms of attached substituents, were tested as molecular fluorescent sensors. These were 2,6-diphenylpyridine (PT-H), 2,6-bis(4-methoxyphenyl)pyridine (PT-OCH_3_), 2,6-bis(4-methylsulphanylphenyl)pyridine (PT-SCH_3_), 2,6-bis(p-tolyl)pyridine (PT-CH_3_), 2,6-bis(4-fluorophenyl)pyridine (PT-F), 2,6-bis(4-cyanophenyl)pyridine (PT-CN), 2,6-bis[4-(trifluoromethyl)phenyl]pyridine (PT-CF_3_) and 2,6-bis(4-methylsulphonylphenyl)pyridine (PT-SO_2_CH_3_). The structures of 2,6-diphenyl pyridine derivatives are shown in Scheme 1. The synthesis pathways of the analysed derivatives are described in detail in the Supplementary Information, which also includes the results of NMR (Figures S1–S16), LC-MS analyses and visualization of the fluorescence of powders of these compounds (Figure S17).

**Scheme 2:**
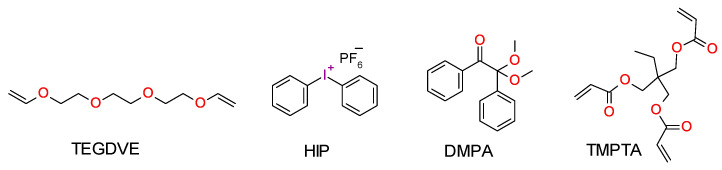
Chemical structures of monomers and photoinitiators.

For the cationic photopolymerization, triethylene glycol divinyl ether (TEGDVE, from Sigma Aldrich) as a model vinyl ether monomer and diphenyliodonium hexafluorophosphate (HIP, from Alfa Aesar) as a photoinitiator were used. For the free-radical photopolymerization, ttrimethylolpropane triacrylate (TMPTA, from Sigma Aldrich) was used as a methacrylate monomer and 2,2-dimethoxy-2-phenylacetophenone (DMPA, from Sigma Aldrich) as a free-radical photoinitiator. The structures of monomers and model photoinitiators are shown in Scheme 2.

### 2.1. Spectral Measurements

The absorption and fluorescence spectra of 2,6-diphenylpyridine derivatives were measured in acetonitrile (from Sigma Aldrich), using the SilverNova spectrometer (StellarNet, Inc., Tampa, FL, USA), in combination with a broadband tungsten–deuterium UV-Vis light source (from StellarNet, Inc., Tampa, FL, USA) and a quartz cuvette with a 1.0-cm optical path. Next, the absorbance data were converted into extinction coefficients, expressed in units [dm^3^ mol^−1^ cm^−1^]. The absorption and fluorescence measurements of 2,6-diphenylpyridine were measured in acetonitrile at 25 °C using 10-mm-thick quartz cells. The fibre-optic cable used for the transmission of light from the measurement site to the spectrometer was made of PMMA optical fibre with a 2-mm core (from Fibrochem, Poland). 

The emissions of the analysed compound 2,6-bis(4-methoxyphenyl)pyridine (PT-OCH_3_) in buffers at different pHs were recorded using the spectrofluorometer FluoroMAX 4Plus with MicroMax Plus (from Horiba, Kioto, Japan), which was used in standard 96-well plates (from Thermo Scientific). 

Fluorescence emission and excitation spectra in different solvents (polarity experiments) were recorded using the Quanta Master™ 40 spectrofluorometer (from Photon Technology International (PTI), currently a part of Horiba, Kioto, Japan) at varied excitation and observation wavelengths in the range of 200–800 nm.

### 2.2. Steady State Photolysis

Steady state photolysis was carried out using the same spectrometer as for the absorption studies. The measurement consisted in the following: cuvettes with the 2,6-diphenylpyridine derivatives in acetonitrile were irradiated by UV-LED 320 nm (UVTOP315-BL-TO39, Roithner Laser Technik GmbH, Wien, Austria) emitting light with wavelength at λ_max_ = 320 nm (~1 mW/cm^2^, current 20 mA), and by UV-LED-300 M300L4 (from Thorlabs Inc., Tampa, FL, USA) emitting light with wavelength at λ_max_ = 300 nm (~26 mW/cm^2^, current 350 mA) for 20 min. The source of light was powered by a DC2200 regulated power supply (from Thorlabs Inc., Tampa, FL, USA). The UV-Vis spectra were recorded with the UV/Vis deuterium-halogen light source SL5 (from StellarNet, Inc., Tampa, FL, USA). The photolysis of 2,6-bis(4-methoxyphenyl)pyridine (PT-OCH_3_) derivatives in the presence of different amounts of iodonium salt (HIP) was determined with the same parameters over 20 min when exposed to UV-LED 320 nm with a maximum emission of 320 nm.

### 2.3. Preparation of Samples for Monitoring the Photopolymerization Processes by FPT 

Samples for the FPT photopolymerization studies were prepared in dark amber glass vials in a dark room by dissolving the photoinitiator diphenyliodonium hexafluorophosphate and each 2,6-diphenylpyridine derivative compound in the monomer in such proportions as to obtain a concentration of 1.0% by weight photoinitiator and 0.1% by weight sensor. The finished samples in the glass vials with the composition were wrapped in aluminium foil and stored in a dark place to protect against accidental exposure to daylight. Prior to the measurement, two drops of the composition were placed at the centre of the microscope slide (75 mm × 25 mm × 1 mm, from Thermo Scientific), equipped with two 0.09-mm thick spacers placed on the sides of the slide. The slide was covered with a second microscope slide to create a sandwich structure. In order to maintain the constant thickness of the composition, the whole was compressed on both sides by clips. The sample prepared in this way was placed into a measuring chamber. Prior to the measurement, the sample was thermostated for a few minutes at the set temperature. For the preparation of thin-layer samples, essential microscope slides with dimensions of 26 mm × 76 mm × 1 mm from Menzel-Glaser, in accordance with ISO 8037/I, were used. A uniform type of self-adhesive paper spacer was used for the spacers.

### 2.4. Monitoring Fluorescence Changes during Photopolymerization

**Figure 1:**
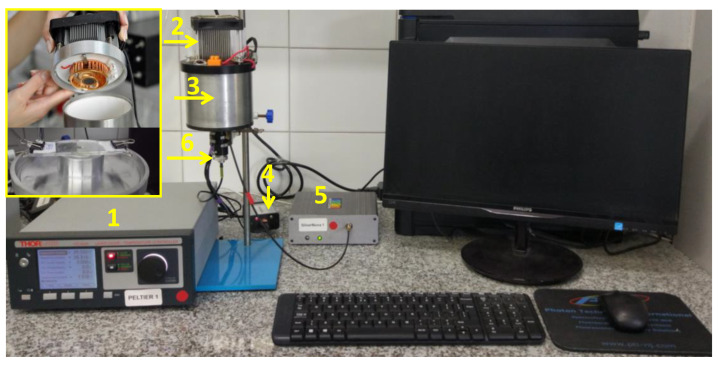
A stationary cure monitoring system was composed of a microcomputer-controlled miniature spectrometer (EPP2000C from StellarNet, Inc., Tampa, FL, USA): 1—thermostat (Thorlabs, Inc., Newton, NJ, USA), 2—Peltier cell with heatsink, 3—measuring chamber, 4—power supply, 5—spectrometer (StellarNet, Inc., Tampa, FL, USA), 6—UV-LED diode.

The apparatus of the Fluorescence Probe Technology was composed of a sample compartment, equipped with a miniature CCD spectrometer (SilverNova from StellarNet, Inc., Tampa, FL, USA), interfaced to a microcomputer for data acquisition, and a UV-LED emitting at wavelength of λ_max_ = 320 nm (UVTOP315-BL-TO39, Roithner LaserTechnik GmbH, Vienna, Austria) incorporated into the sensor head. FPT photopolymerization reaction studies were carried out under controlled, thermostatic conditions. For this purpose, a measuring chamber, impermeable to daylight, was specially designed and equipped with a thermostatic head, with high accuracy (±0.1 °C), which maintained the set temperature inside the chamber using an electronically controlled Peltier cell as a heat pump. Depending on the temperature difference between the inside of the chamber and the surroundings, the cell carried heat outside or inside the chamber. The measuring head, chamber bottom and thermostatic head were made of aluminium, and the walls were made of a piece of 4-inch stainless steel pipe with an external diameter of 101.6 mm and a wall thickness of 2 mm. The interior of the chamber was additionally covered with a layer of Teflon, which served as a heat insulator. A measuring head with a UV-LED was mounted at the bottom of the chamber, where a sample of the composition directly adhered, whose spot (about 5 mm in diameter) illuminated the tested thin-film sample. A PMMA optical cable with a core diameter of 2 mm was used to transfer fluorescent light from the measurement site to the spectrometer. The UV LED was powered by a 20-mA direct current from a stabilised current source. The main advantage of this measuring chamber was the high intensity of the excitation light from the same type of UV-LEDs and the small scattering of the excitation light compared to previous measuring systems [22]. This was due to the elimination of one of the fibre-optic cables and the incorporation of the UV-LED directly into the chamber head. The appearance of the apparatus for monitoring the kinetics of photopolymerization processes was similar to those described earlier [50], albeit modernised by means of a thermostat system that guaranteed the stability of environmental conditions during photopolymerization. Free-radical and cationic photopolymerization processes were carried out at an ambient temperature of 25 °C using the ITC4020 thermostat (from Thorlabs Inc., Newton, NJ, USA). A photograph of the measurement system is shown in Figure 1. 

## 3. Results and Discussion

### 3.1. Spectroscopic Properties of 2,6-Diphenylpyridine Derivatives

**Figure 2:**
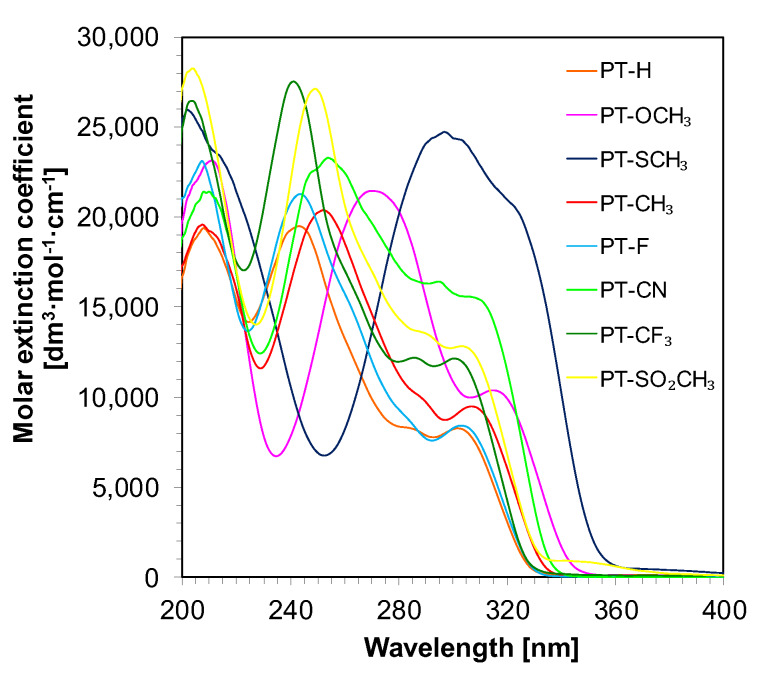
UV-visible absorption spectra of the 2,6-diphenylpyridine derivatives in acetonitrile.

The most important parameters describing the spectroscopic properties of molecular probes are absorbance, the molar extinction coefficient, fluorescence and Stokes shift. The analysis of the electronic absorption spectra of 2,6-diphenylpyridine derivatives clearly indicates the presence of two main bands, whose maxima are located in the UV-C range between 220–280 nm for the first band, and in the UV-B range above 300 nm for the second band (Figure 2, Table 1). The shortest wavelength bands are attributed to the π→π* transitions, whereas the long wavelength bands, are attributed to the n→π* transitions.

**Figure 3:**
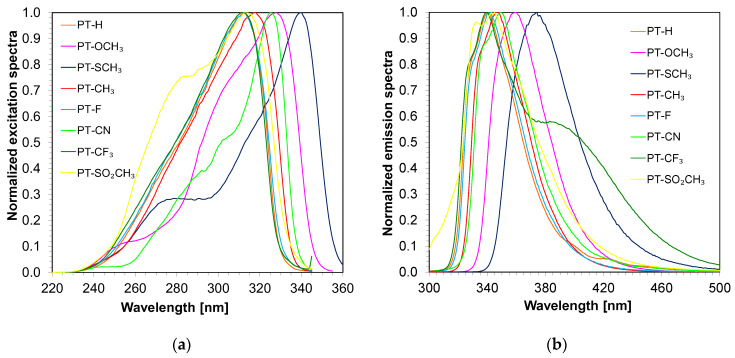
(**a**) Normalized excitation spectra of the 2,6-diphenylpyridine derivatives in acetonitrile; (**b**) Normalized emission spectra of the 2,6-diphenylpyridine derivatives in acetonitrile; λ_ex_ = 290 nm is the excitation wavelength, used to obtain emission spectra.

In order to determine the Stokes shift, the emission and excitation spectra of the investigated compounds were measured. The calculated Stokes shift ranged from 2116 cm^−1^ for 2,6-bis(4-cyanophenyl)pyridine (PT-CN), to 2963 cm^−1^ for 2,6-bis(4-methylsulphonylphenyl)pyridine (PT-SO_2_CH_3_). In addition, compounds with electron-donating substituents, i.e., SCH_3_, OCH_3_ and CH_3_, show a bathochromic shift of both excitation and emission spectra in the direction of longer waves compared to 2,6-diphenylpyridine (PT-H) (Figure 3). For example, the wavelength for the maximum intensity of the emission spectrum for the 2,6-bis-(4-methylsulphanylphenyl)pyridine (PT-SCH_3_) is located at 374 nm, so it is 33 nm higher than the wavelength for the maximum intensity of the emission spectrum for the basic compound 2,6-diphenylpyridine (PT-H) with maximum emission located at 341 nm. All spectrum data are presented in Table 2 (the visualization of the fluorescence of the pyridines in solution Figure S18).

**Figure 4:**
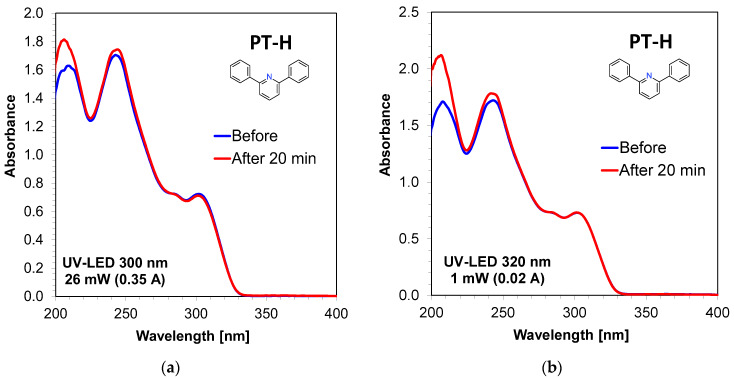
Photolysis of 2,6-diphenylpyridine (PT-H, concentration: 8.83 × 10^−^^5^ [mol/dm^3^]) in ACN during 20 min: (**a**) under 300 nm (26mW/cm^2^); (**b**) under 320 nm (1mW/cm^2^).

Due to the fact that photopolymerization processes are very fast, they require continuous exposure to light of a certain length; therefore, an analysis of the photostability of the actual derivatives was undertaken. The stability of the 2,6-diphenylpyridine derivatives was tested by measuring the absorbance response during 20 min in acetonitrile under irradiation by UV-LEDs with maximum emission at 300 nm (intensity of irradiation 26 mW/cm^2^) and at 320 nm (intensity of irradiation 1 mW/cm^2^) (Figure 4). The absorption characteristics were continuously monitored and recorded at set time intervals across the entire spectrum. The results indicated that the 2,6-diphenylpyridine derivatives could be used as sensors because of their good stability with no solids precipitated during irradiation. Thus, the probe can be used to monitor kinetic parameters during photopolymerization processes in real time. Other spectra are presented in the Supplementary Information (Figures S19–S31).

**Figure 5:**
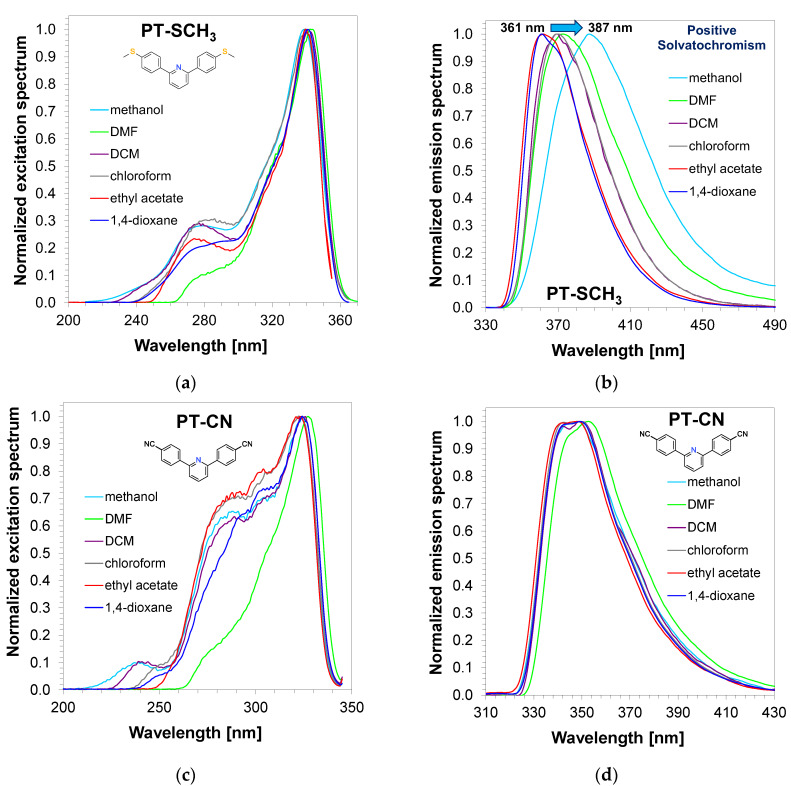
Spectroscopic properties of diphenylpyridine derivatives in solvents of different polarity: (**a**) normalised excitation spectrum for 2,6-bis(4-methylsulphanylphenyl)pyridine (PT-SCH_3_); (**b**) normalised emission spectrum for 2,6-bis(4-methylsulphanylphenyl)pyridine (PT-SCH_3_); (**c**) normalised excitation spectrum for 2,6-bis(4-cyanophenyl)pyridine (PT-CN); (**d**) normalised emission spectrum for 2,6-bis(4-cyanophenyl)pyridine (PT-CN).

Due to the fact that FPT technology is based on the analysis of changes in the fluorescence sensor emission spectra during the photopolymerization process, it was decided to proceed to the next research stage related to the analysis of the sensitivity of the developed derivatives to changes in environmental polarity. For this purpose, two different types of 2,6-diphenylpyridine derivatives were selected in order to study the effect of changes in solvent polarity on their emission and excitation spectra. The first investigated sensor was 2,6-bis(4-methylsulphanylphenyl)pyridine (PT-SCH_3_), which has an electron-donating substituent in the diphenylpyridine framework. The second derivative was 2,6-bis(4-cyanophenyl)pyridine (PT-CN) with an electron-withdrawing substituent in its structure. The figures of the emission and excitation spectra of the analysed compounds are presented in Figure 5. In both cases, during changes in the polarities of the solvents, no significant changes of the position of the maximum in their excitation spectra were observed. Nevertheless, this was not the case when the emission spectra of the PT-SCH_3_ compound were analysed depending on solvent polarity. Examining the data summarised in Table 3 and Figure 5, it was seen that the maximum of fluorescence only changed for the PT-SCH_3_ derivative. The position of maximum fluorescence intensity ranged from 361 nm to 387 nm and highly depended on the polarity of the solvents. This compound, which has an electron-donating substituent into 2,6-diphenylpyridine moiety, exhibits a shift in emission spectra as solvent polarity increases. 

Based on the obtained spectroscopic data, it can be seen that both molecules differ in the amount of dipole moment excited. More specifically, the derivative of PT-SCH_3_ shows a significant change in dipole moment at the moment of electron excitation, while PT-CN shows only a small change. Since the polar solvent interacts more strongly with the molecule, and if there is a rapid change in its dipole moment, for example, during excitation, the solvent molecules remain not optimally aligned to the PT-SCH_3_ molecule. Solvent relaxation reduces the energy of the excited state (stabilizes it), and, hence, the observation of such a significant change in the photons’ energy is emitted by PT-SCH_3_ in polar solvents. Therefore, the observed differences between PT-SCH_3_ and PT-CN result from different dipole moments during the excitation of these molecules. PT-SCH_3_ is generally more polarized than the other derivatives; therefore, the transitions between its states have the least energy.

**Figure 6:**
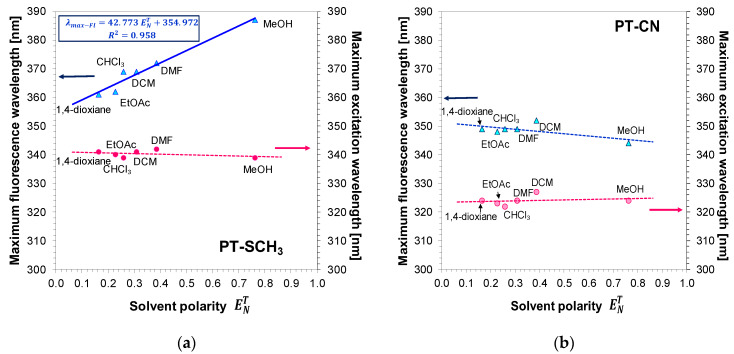
Dependence of the location of the maximum intensity of the emission and excitation spectra for (**a**) 2,6-bis(4-methylsulphanylphenyl)pyridine (PT-SCH_3_) and (**b**) 2,6-bis(4-cyanophenyl)pyridine (PT-CN) on the polarity of the solvents (scale $\;E_{N}^{T}$).

Therefore, the correlation between solvent polarity in the Dimroth–Reichardt solvent scale E_T_(30) and maximum fluorescence wavelength for 2,6-bis(4-methylsulphanylphenyl)pyridine was examined, and a linear relationship with a good correlation coefficient (R^2^) was obtained (R = 0.96) (Figure 6). The positive slope of the linear correlations between the maxima of fluorescence wavelength positions and solvent polarity indicates that the sensor exhibits positive solvatochromism. Moreover, larger slope values in the case of fluorescence data than in the case of absorption data indicate higher polarity of the excited state of the probe than the polarity of the ground state. Based on these measurements, we can confidently predict that the PT-SCH_3_ compound will be sensitive to changes of polarity during the process of photopolymerization. This is due to the fact that, as the photopolymerization of the monomer progresses, the polarity of the system decreases, because the monomer double bonds, which are more polarised, are converted into single bonds in the polymer, which are less polarised. Thus, the polarity of the environment is reduced. In contrast, 2,6-bis(4-cyanophenyl)pyridine (PT-CN), with an electron-withdrawing substituent in the diphenylpyridine framework, is not sensitive to changes in polarity and is not suitable as a sensor sensitive to changes in polarity (Figure 5 and Figure 6).

The polarization of the pyridine derivatives with electron-donor groups is higher than that of the derivatives with electron-withdrawing substituents. This phenomenon causes the ICT transition energy to be lower (the higher the polarization of the molecule, the lower the energy of the electronic transition). Besides, the change in the dipole moment of the molecule during the excitation can be significant, which in turn causes a reduction in the energy of the excited state (solvent relaxation) and thus a smaller energy of the radial return of the molecule to the ground state.

Consequently, the polarization of the molecule with two electron-donor groups at the ends and an electron-acceptor group in the centre is more significant than when the molecule is equipped with a bare phenyl ring—hence, the observed effects appear.

### 3.2. Monitoring of Free-Radical Photopolymerization Using 2,6-Diphenylpyridine Derivatives

Subsequently, studies on the applicability of 2,6-diphenylpyridine derivatives as molecular fluorescent probes for monitoring the free-radical photopolymerization of acrylate monomers (TMPTA) using Fluorescence Probe Technology were carried out [52,53]. During the photopolymerization process, polarity changed and viscosity dramatically increased because the liquid monomer transforms into a solid polymer and, as mentioned above, polarity generally decreases. Therefore, fluorescent molecular sensors should respond to a change in viscosity and/or polarity by changing the characteristics of the fluorescence spectra. 

**Figure 7:**
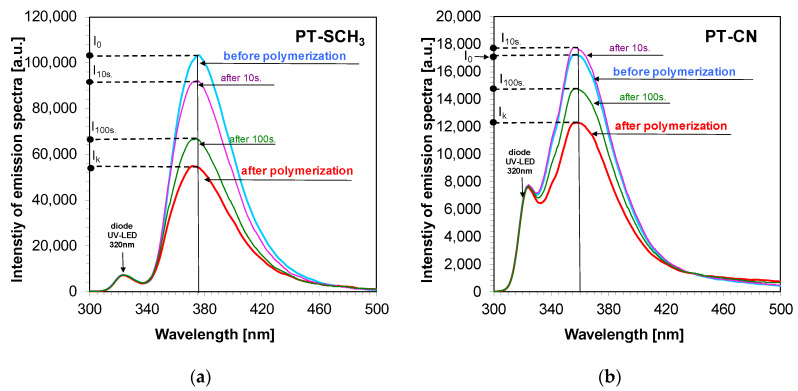
Changes of fluorescence spectra during free-radical photopolymerization of the TMPTA monomer under irradiation of 320 nm: (**a**) 2,6-bis(4-methylsulphanylphenyl)pyridine (PT-SCH_3_); (**b**) 2,6-bis(4-cyanophenyl)pyridine (PT-CN).

The free-radical photopolymerization process was carried out in UV-LED irradiation with a maximum emission of 320 nm. Generally, the bathochromic or hypsochromic shifts of emission spectra from fluorescent probes depend on the nature and character of the probe, as observed during the free-radical photopolymerization process [44]. The following figure, Figure 7, indicates the monitoring of the changes of the fluorescence spectra of selected 2,6-bis-phenylpyridine compounds (PT-SCH_3_ and PT-CN) during the free-radical photopolymerization of TMPTA monomers under irradiation of 320 nm. Information on other potential sensors can be found in the Supplementary Materials (Figures S32–S38). During the photopolymerization process, a decrease in fluorescence intensity was generally observed for these probes. A slight shift of emission spectra (Δλ_max_ = 5nm), however, was obtained for 2,6-bis(4-methylsulphanylphenyl)pyridine (PT-SCH_3_) (Figure 7a).

**Figure 8:**
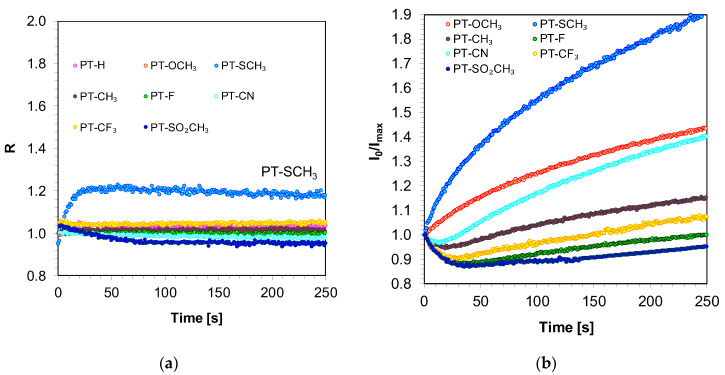
Monitoring the free-radical photopolymerization of the TMPTA monomer under 320 nm by FPT, using the 2,6-bis(4-methylsulphonylphenyl)pyridine as fluorescent sensor and (**a**) the R parameter and (**b**) the I_0_/I_max_ parameter.

In consequence, the fluorescence intensity ratio (R) was applied as an indicator of polymerization progress. R was defined as the ratio of fluorescence intensity at a shorter wavelength (λ_1_) to the intensity at a longer wavelength (λ_2_), both located on the opposite sides of the fluorescence spectrum maximum. The monitoring wavelengths were determined individually for each probe from the probe fluorescence spectrum taken before monomer polymerization. Thus, the defined R values started from 1 and increased when the fluorescence spectrum shifted to shorter wavelengths or stayed constant when the spectrum did not shift. Figure 8a shows the effect of the measured free radical photopolymerization in real time, using the fluorescence intensity ratio (R) as progress indicator. It can be clearly seen that, depending on the type of substituents on the 2,6-diphenylpyridine moiety, the compounds either shifted or not shifted their fluorescence spectrum before and after photopolymerization, which is reflected in the ratio span between the uncured and cured states of the composition (Figure 8a). From among the probes studied, the sensor PT-SCH_3_ can be used to monitor the kinetics of the free-radical photopolymerization process using the increase of the ratio (R) with the progress of monomer polymerization. For the rest of the pyridine derivatives, this was impossible because the emission spectra of these compounds did not change position during the polymerization (Figure 8 and Table 4).

Therefore, in the case of other 2,6-diphenylpyridine derivatives, the normalised intensity (I_0_/I_max_) parameter can be applicable to the curing processes under stationary measurement conditions. Figure 8b shows the kinetic profiles of the photopolymerization process, obtained using normalised fluorescence intensity (I_0_/I_max_) as the polymerization progress indicator. All derivatives except one, 2,6-diphenylpyridine (PT-H), exhibit a large enough change in the parameter I_0_/I_max_ during monomer polymerization to be applied as fluorescent sensors for monitoring free-radical photopolymerization. 

In conclusion, based on these experiments, it was shown that the changes in emission spectra for PT-SCH_3_ are caused by changes in the polarity and viscosity of the surrounding environment. Conversely, for the rest of the 2,6-diphenylpyridine derivatives, changes in emission spectra are dictated only by changes in viscosity, as shown for the example of the PT-CN derivative in the spectroscopic experiment with solvents with different polarity. Therefore, the effect of substituents on the sensitivity of the 2,6-diphenylpyridine derivatives as sensors during free-radical photopolymerization is significant. Nevertheless, depending on the type of substituents, either the fluorescence intensity ratio (R) or the normalised intensity (I_0_/I_max_) can be used as quantitative indicators of the free-radical polymerization progress. 

### 3.3. Monitoring of Cationic Photopolymerization Using 2,6-Diphenylpyridine Derivatives—Is It Possible?

**Figure 9:**
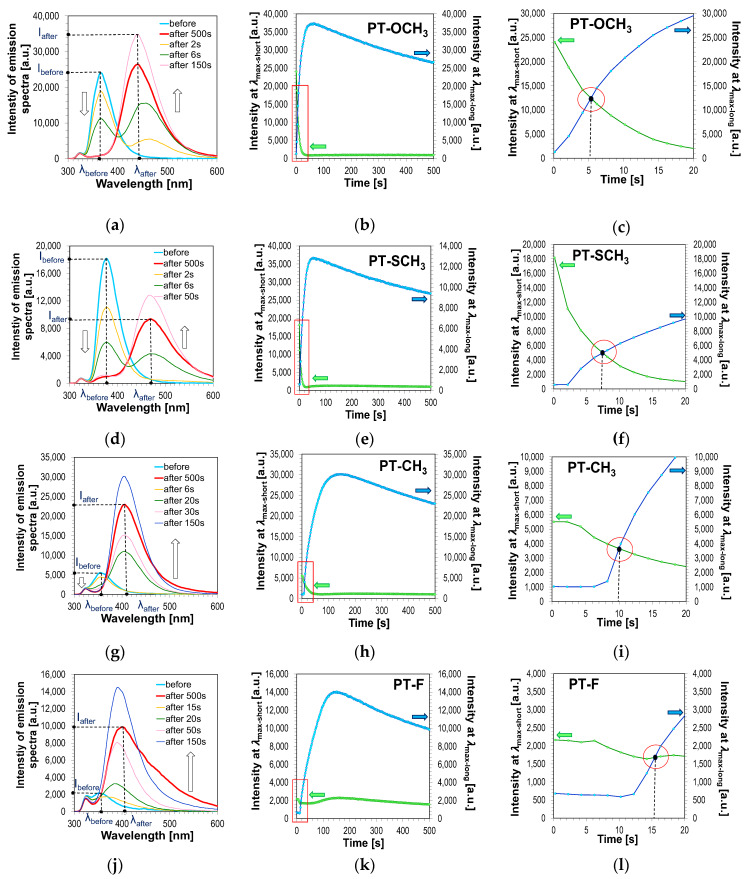
(**a,d,g,j**) Changes of fluorescence spectra of the 2,6-diphenylpyridine derivatives during cationic photopolymerization of TEGDVE monomer under irradiation at 320 nm; (**b,c,e,f,h,i,k,l**) monitoring cationic photopolymerization of TEGDVE monomer under 320 nm by FPT, using the 2,6-diphenylpyridine derivatives as the fluorescent sensors.

**Figure 10:**
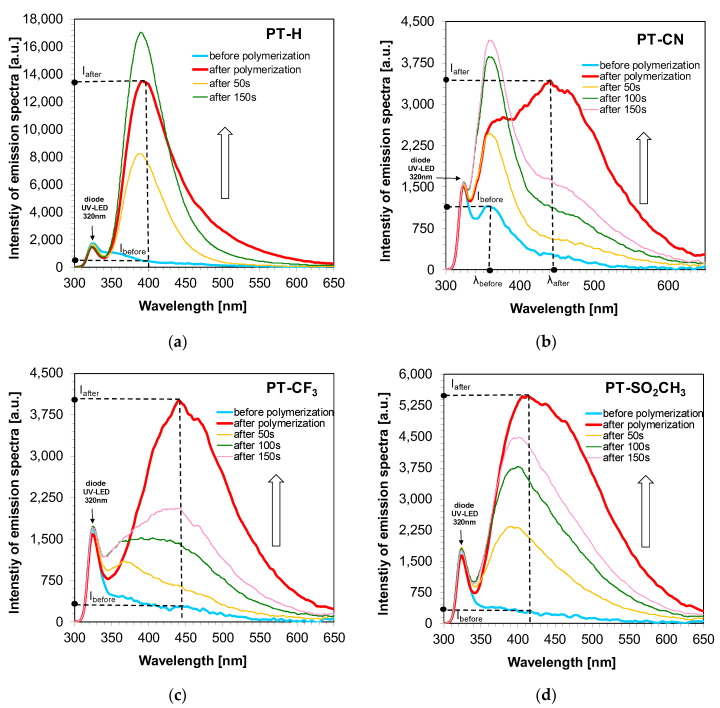
Changes of fluorescence spectra during cationic photopolymerization of TEGDVE monomer under irradiation 320 nm for (**a**) PT-H sensor; (**b**) PT-CN sensor; (**c**) PT-CF_3_ sensor; (**c**) PT-SO_2_CH_3_ sensor.

Similar measurements of the applicability of 2,6-diphenylpyridine derivatives as potential fluorescent molecular sensors for monitoring the kinetics of the cationic photopolymerization processes of vinyl monomer (TEGDVE) were carried out. As with free-radical photopolymerization, the compositions were irradiated with UV-LED with a maximum emission of 320 nm. Theoretically, every process that causes a change in the system’s polarity or micro-viscosity should be able to be monitored by fluorescent sensors. Depending on the type of process and monitoring parameters, however, the relevant structure and characteristics of the sensors are required. This is exactly what happened during the monitoring of cationic photopolymerization with 2,6-diphenylpyridine derivatives. The behaviours of 2,6-diphenylpyridine derivatives during the cationic photopolymerization of the TEGDVE monomer turned out to be different from the behaviour of the same compounds during the free-radical photopolymerization of the acrylate monomer TMPTA studied previously. Upon comparison of the fluorescence spectra of the 2,6-diphenylpyridine derivatives at different irradiation times, it was noted that the fluorescence intensity of all investigated compounds decreased significantly during cationic photopolymerization. For compounds 2,6-bis(4-methoxyphenyl)pyridine (PT-OCH_3_), 2,6-bis(4-methylsulphanylphenyl)pyridine (PT-SCH_3_), 2,6-bis(p-tolyl)pyridine (PT-CH_3_) and 2,6-bis(4-fluorophenyl)pyridine (PT-F), the following was observed: a dramatic decrease in fluorescence intensity at maximum emission fluorescence located at shorter wavelengths (λ_max-short_) below 400 nm, and simultaneous appearance and increase of fluorescent intensity of a completely new fluorescence band at longer wavelengths (λ_max-long_) (Figure 9a,d,g,j). Moreover, during the cationic photopolymerization process for the 2,6-diphenylpyridine (PT-H) and for compounds with electron-withdrawing substituents, i.e., PT-CF_3_ and PT-SO_2_CH_3_, an increase of the fluorescence intensity at longer wavelengths was observed. As these compounds exhibit very weak fluorescence prior to the cationic photopolymerization of vinyl monomers, the fluorescent spectrum in shorter wavelengths was not studied. Only when the photocurable samples were exposed under UV light was there a significant increase in fluorescence intensity (Figure 10) observed. This clearly indicates that substitution in the phenyl rings at the *para-* position of the 2,6-diphenylpyridine moiety plays an important role in the generation of a push-pull effect in the excited states of the compounds. The highest sensitivity was achieved in the case of PT-OCH_3_ containing an electron-donating methoxy group in the *para-* position of the phenyl rings. This is well illustrated in Table 5, showing the effect of Hammett substituent constants (σ_p_) on the speed of the decrease of fluorescent intensity localised at shorter maximum emissions of the 2,6-diphenylpyridine derivatives.

At this stage of the research, the question arose: is it possible to monitor the kinetics of the cationic photopolymerization process using the FPT method with pyridine derivatives? From a practical point of view, the compound used as a fluorescent sensor to track changes in polymerization kinetics should only be sensitive to changes associated with this process. Thus, for example, it should be sensitive to changes in polarity and/or viscosity in the entire monomer conversion range. Based on the recorded data, however, it can be seen that when the light is switched on to initiate the photopolymerization process, the fluorescence spectrum localised at the UV-A region of the sensors (PT-OCH_3_), (PT-SCH_3_), (PT-CH_3_) and (PT-F) decreases dramatically. Moreover, for these compounds, the simultaneous appearance of a completely new fluorescence band located in a more extended wavelength range is observed (Figure 9b,c,e,f,h,i,k,l).

Therefore, it is crucial to answer the question of what it is we are monitoring using this group of compounds during the cationic polymerization process. Based on earlier research, it is known that the developed derivatives exhibit photostability when exposed to both 320 nm light and UV-C light of 300 nm. Therefore, the likelihood of photobleaching has been excluded. During the photocleavage process of iodonium salt, at the stage of initiation of cationic polymerization, strong protic acids are generated. It was likely that the monitored response of the analysed sensors relates to the online monitoring of the strong acid generation rate at the initiation stage. Consequently, the responses of the sensors do not track the consumption of monomers or do not monitor increase the degree of conversion and progress of polymerization. In order to carry out more extensive research on the effects of the environment on the spectroscopic response of 2,6-diphenylpyridine derivatives, the effect of pH on the fluorescence characteristics of the developed derivatives was investigated.

### 3.4. Applicability of 2,6-Diphenylpyridine Derivatives as Fluorescent pH Sensors

**Figure 11:**
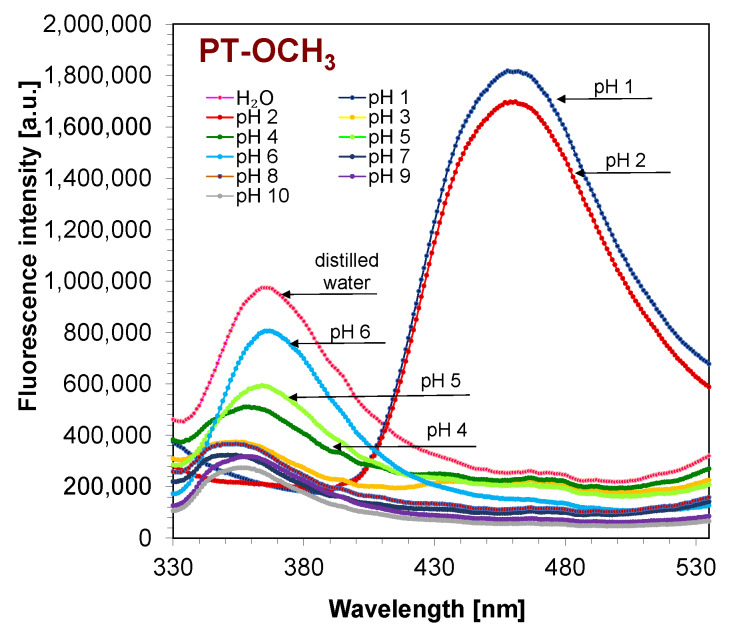
Comparison of fluorescence spectrum maximum location for compound PT-OCH_3_ in acetonitrile and pH in a range of 1–10.

**Figure 12:**
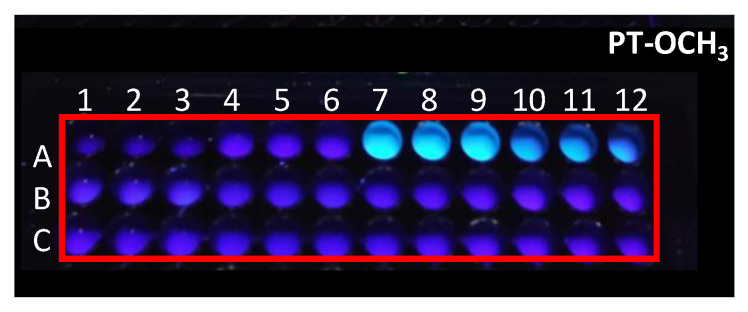
Dependence of the emission spectra of the PT-OCH_3_ on different pH environments. Photograph illustrates the fluorescence intensity of the PT-OCH_3_ compound in acetonitrile under a UV-LED lamp with a maximum emission of 365 nm in an environment of various pH values: A1–A3 acetonitrile; A4–A6 H_2_O; A7–A9 pH 1; A10–A12 pH 2; B1–B3 pH 3; B4–B6 pH 4; B7–B9 pH 5; B10–B12 pH 6; C1–C3 pH 7; C4–C6 pH 8; C7–C9 pH 9; C10–C12 pH 10.

The next step was to test the applicability of the new 2,6-diphenylpyridine derivatives for monitoring pH changes. To this end, 2,6-bis-(4-methoxyphenyl)pyridine (PT-OCH_3_), which has an electron-donating substituent located in the phenyl rings, was selected. As shown in Figure 11, the maximum emission wavelength of 2,6-bis-(4-methoxyphenyl)pyridine (PT-OCH_3_) is located at 354 nm for pH 7.0, with an intensity of 3.23 × 10^5^ [a.u.]. Upon decreasing the pH from 7.0 to 1.0, however, the emission spectrum of 2,6-bis-(4-methoxyphenyl)pyridine, which is 104 nm, bathochromically shifted to 458 nm, which is a sixfold increase of the intensity of fluorescence, accompanied by the gradual disappearance of the peak of the emission located at 354 nm (Figure 11 and Table 6). The large bathochromic shift of the maximum of the fluorescence spectrum was attributed to the enhanced ICT effect because of the H^+^-binding-induced enhancement of the electron-withdrawing ability of pyridine. The obtained red shift of the spectrum of PT-OCH_3_ provided a good opportunity to achieve ratiometric detection in the acidic region. The large shift in the fluorescence emission peak caused a variation in emission colour. With decreasing pH, the colour of the PT- OCH_3_ sensor in the solution changed from UV to blue (Figure 12). Therefore, the 2,6-bis-(4-methoxyphenyl)pyridine (PT- OCH_3_) can act as a ‘‘naked-eye’’ fluorescence sensor for acidic pH environments.

The above results allowed us to propose a mechanism of interactions between the sensor and the acidic environment properties. We postulate that, under acidic conditions, the pyridine N atom is protonated in the form of NH^+^. Protonated pyridine acts as an electron acceptor and the phenyl rings with electron-donating substituents act as electron donors. This mechanism can be responsible for the sensors’ capacity for real-time pH monitoring in a strong acidic region. In order to examine whether these pH-dependent emission ratio changes of the 2,6-bis-(4-methoxyphenyl)pyridine (PT-OCH_3_) and 2,6-bis(4-methylsulphanylphenyl)pyridine (PT-SCH_3_) are useful for monitoring cationic photopolymerization, and especially the process of decomposition of iodonium salt with the generation of a strong protic acid, the relevant experiments were carried out. 

**Figure 13:**
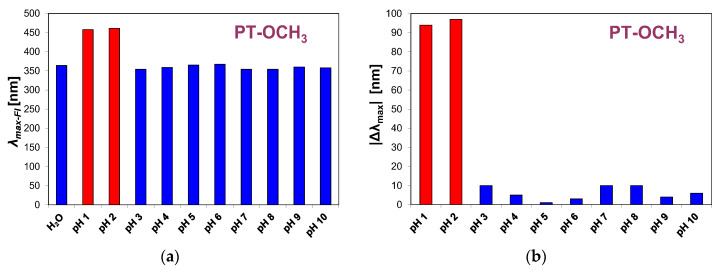
(**a**) Position of maximum fluorescence intensity for compound PT-OCH_3_ in in distilled water and pH in a range of 1–10. (**b**) Difference between the position of the maximum fluorescence of the PT-OCH_3_ compound in the reference sample (in distilled water) and the maximum of fluorescence in buffers at different pH.

Most fluorescence pH probes are practical in a near-neutral pH region, and probes for detecting the pH changes in the acidic range are limited [55]. Moreover, fluorescence pH sensors are still subject to limitations based on their limited photostability. Thus, developing photostability fluorescent probes possessing large Stokes’ shift for monitoring acid pH is of great importance. The investigated pyridines meet these requirements due to their photostability, the significant bathochromic shift around 90 nm (Figure 13), and their good solubility in non-polar as well as polar environments. Such properties enable the 2,6-diphenylpiridine derivatives to work as fluorescent acidic pH sensors in the solution state.

### 3.5. 2,6-Diphenylpyridine as Photometric Fluorescent Sensor for the Detection, Determination of Efficiencies and Quantification of Superacid Generation upon Exposure of Iodonium Photoinitiator to Light

**Figure 14:**
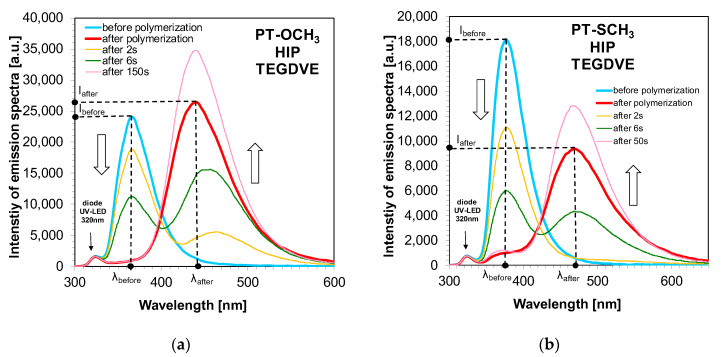
Changes of fluorescence spectra during cationic photopolymerization of TEGDVE monomer under irradiation at 320 nm for (**a**) PT-OCH_3_ sensor and (**b**) PT- SCH_3_ sensor.

**Figure 15:**
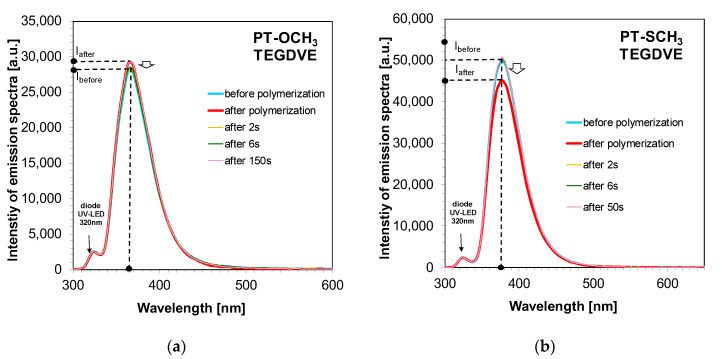
Changes in emission spectra during irradiation at 320 nm of the system containing the TEGDVE monomer without the addition of a HIP photoinitiator and (**a**) the PT-OCH_3_ sensor and (**b**) the PT- SCH_3_ sensor.

**Figure 16:**
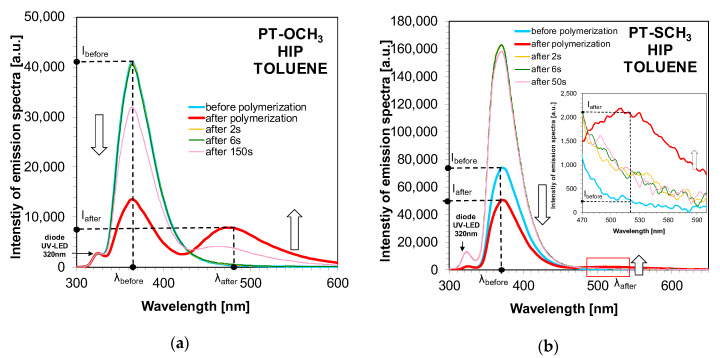
Changes in emission spectra during irradiation at 320 nm of the system containing the toluene, HIP photoinitiator and (**a**) the PT-OCH_3_ sensor and (**b**) the PT- SCH_3_ sensor.

Encouraged by these interesting results, a series of experiments were subsequently carried out, which should explain the appearance of a second fluorescence band during the cationic photopolymerization process of the TEGDVE vinyl monomer. For this purpose, three different compositions were prepared and measured by spectroscopic techniques. The first sample was the standard photocurable composition based on fluorescent sensors, a photoinitiator and vinyl monomers (sensor/HIP/TEGDVE); the second sample had sensors dissolved in vinyl monomer (sensor/TEGDVE); and the third sample was based on the investigated sensor with the cationic photoinitiator dissolved in the non-reactive and non-polar solvent (sensor/HIP/toluene). For this purpose, three different compositions were prepared: a standard photocurable composition based on fluorescent sensors, a photoinitiator and vinyl monomer (sensor/HIP/TEGDVE), a probe/photoinitiator (HIP) and another probe/photoinitiator (HIP)/toluene. The first composition probe/HIP/TEGDVE is a standard composition used for monitoring the measurements of the kinetics of the cationic photopolymerization process. The second composition did not contain a photoinitiator, iodonium salt, and thus the composition could not polymerize. The third composition contained the HIP photoinitiator but did not contain a monomer other than a solvent toluene, which, like the TEGDVE monomer, is non-polar. In the final case, the composition cannot be polymerized due to the lack of a monomer. All three types of compositions were irradiated with a UV-LED diode with a maximum emission of 320 nm (1mW/cm^2^) for 500 seconds. In the case where no HIP photoinitiator was used, no significant changes in emission spectra were observed for the PT-OCH_3_ and PT-SCH_3_ probes when irradiating the composition with a 320 nm UV-LED diode (Figure 14,Figure 15,Figure 16).

**Scheme 3:**
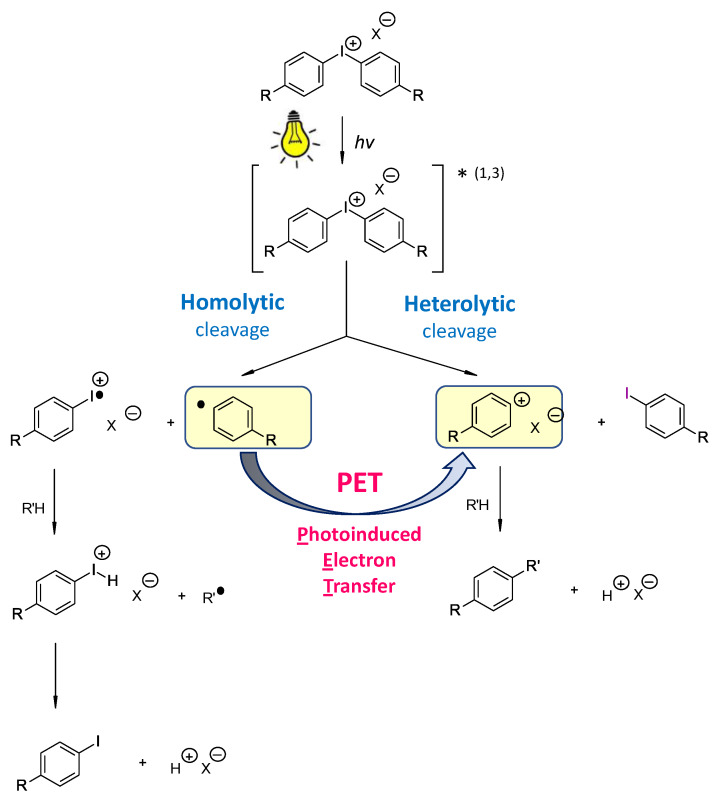
Photolysis of diphenyliodonium salt as a typical example of onium salt.

When irradiating a composition consisting of PT-OCH_3_/HIP/toluene and PT-SCH_3_/HIP/toluene, a decrease in fluorescence intensity at one wavelength and the appearance of a second fluorescence band at a longer wavelength were observed. During this process, the polarity and viscosity of the system did not change. Therefore, changes in emission spectra had to be caused by the release of a strong protic acid from the diphenyliodonium hexafluorophosphorane (HIP) photoinitiator. During exposure, the photoinitiator photocleaved according to Scheme 3:

Onium salts are degraded via homolytic or heterolytic cleavage upon light irradiation. During the process of irradiation, a strong protic acid (HX) is generated through the reaction of a proton source (R-H) with the species generated by homolytic (path A) or heterolytic (path B) cleavage. The generated protic acid is responsible for the protonation of the probes PT-OCH_3_ and PT-SCH_3_.

**Figure 17:**
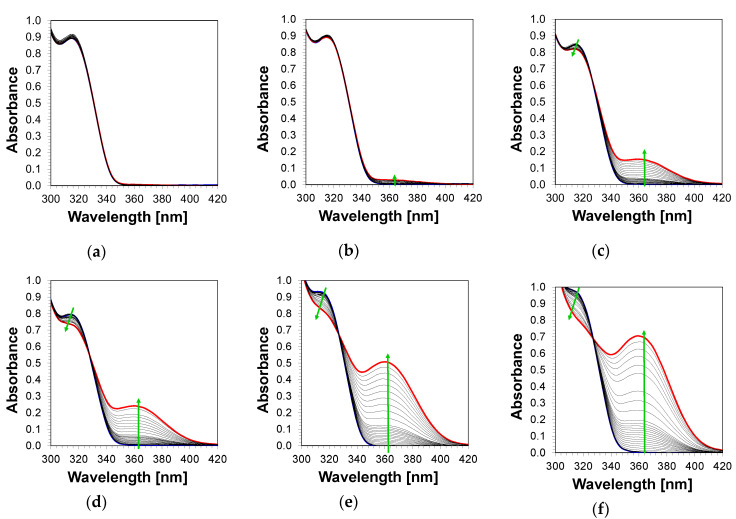
Photolysis of HIP/PT-OCH_3_ in acetonitrile upon exposure to LED @ 320 nm (1mW/cm^2^) in a molar ratio: (**a**) 0.5:1; (**b**) 1:1; (**c**) 5:1; (**d**) 10:1; (**e**) 20:1; (**f**) 30:1.

**Figure 18:**
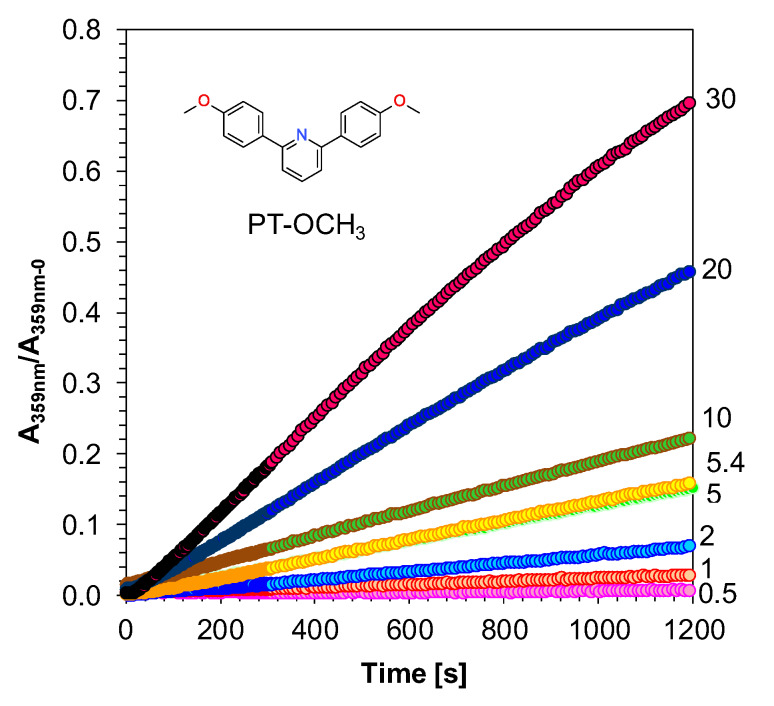
Profiles illustrating changes in absorbance over time of PT-OCH_3_ at 320 nm (1 mW/cm^2^) upon adding various amounts of HIP photoinitiator using the A_359nm_/A_359nm-0_ parameter.

Moreover, changes in absorbance spectra during photolysis were mainly due to the release of a strong protic acid from the HIP photoinitiator. These studies allow the determination of efficiencies of superacid generation by cationic photoinitiators. Therefore, in order to confirm the sensitivity of new fluorescent probes PT-OCH_3_ and PT-SCH_3_ on acidic environment properties, photolysis of the PT-OCH_3_ compound in acetonitrile with different amounts of cationic photoinitiator was performed. The probe/HIP molar ratios were 0.5:1; 1:1, 2:1, 5:1, 5.4:1, 10:1, 20:1 and 30:1, respectively. The samples were irradiated with a UV-LED diode with a maximum emission of 320 nm, so that the photolysis process was carried out under the same conditions as the cationic photopolymerization process of the TEGDVE monomer. The spectra of the photolysis examples are presented in Figure 17, and the other spectra are presented in the Supplement (Figures S39–S40). During photolysis, in cases where half the amount of photoinitiator was added to the probe solution, no significant changes in absorption spectra were observed (Figure 17a). In the case of the PT-OCH_3_ and HIP compositions in a 1:1 molar ratio, it was observed that a band appeared during the photopolysis process at a maximum of about 359 nm. Conversely, at the molar ratio of HIP/probe 5.4, i.e., as in the measurements of the photopolymerization process of the TEGDVE monomer using Fluorescence Probe Technology, a significant increase in bandwidth at 359 nm was observed (Figure S40). In turn, the 30-fold excess of the HIP photoinitiator caused an even greater bandwidth increase at 359 nm, which clearly indicates the release of a strong protic acid derived from the HIP photoinitiator (Figure 18).

With decreasing pH and as the process of photocleavage of onium salt progresses, the fluorescence emission spectra, as well as absorption spectra of the sensors exhibited bathochromic shifts, resulting in large changes in emission ratios. It was found that the sensors are valuable for determining the progress of decomposition of cationic photoinitiators based on iodonium salts. The response of these sensors to process the photocleavage of photoinitiators relies on the H^+^ binding with the N of pyridine and the induced enhancement of the ICT process.

## 4. Conclusions

In summary, new fluorescent molecular sensors based on 2,6-diphenylpyridine were designed and synthesised by introducing electron-donating or electron-withdrawing groups into the diphenylpyridine framework. The synthesis route of pyridines was a convenient and one-step synthesis via a bis Suzuki–Miyaura (SM) cross-coupling reaction. The applicability of a series of pyridine derivatives as molecular fluorescent sensors for monitoring free-radical and cationic photopolymerization by Fluorescence Probe Technology (FPT) was studied. From among the probes studied, only the 2,6-diphenylpyridines containing an electron-donating substituent at the phenyl rings shift their fluorescence spectrum to shorter wavelengths upon monomer polymerization far enough to enable the use of the fluorescence intensity ratio (R) as the free-radical polymerization progress indicator. The R span of the remaining 2,6-diphenylpyridine derivatives is too small for practical applications; with these compounds, the normalised fluorescence intensity (I/I_0_), which is less versatile than R, has to be used instead. In comparison to the 2-amino-4,6-diphenyl-pyridine-3-carbonitrile derivatives presented in a previous paper [49], the 2,6-diphenylpyridines are colorless compounds, which makes an unquestionable advantage. Therefore, 2,6-diphenylpyridine derivatives do not affect the colour of the formulations before and after the photopolymerization processes. They can be applied to photo-curing coating materials, such as varnishes, for which obtaining a highly transparent effect is extremely important from an industrial point of view. Consequently, the 2,6-diphenylpyridine derivatives can be used as fluorescent molecular sensors to monitor the progress of photopolymerization processes during production (i.e., on-line) without worrying about the effect on the final properties of the coating.

Nevertheless, fluorescence measurements during cationic photopolymerization indicated that all 2,6-diphenylpyridine derivatives could serve as sensitive sensors for pH measuring in acidic regions through colorimetric and fluorimetric changes. The significant bathochromic shift (around 90 nm), good solubility in non-polar environmental, the sensitivity in the acidic range and extremely short response time under extreme acidic conditions of the probe makes it an interesting pH sensor. It is the belief of the authors that it will be greatly beneficial to study the effectiveness of the photodecomposition processes of cationic photoinitiators in order to evaluate the best molecule for this purpose. Therefore, 2,6-diphenylpyridine derivatives are a promising starting point for the development of efficient and versatile colorimetric and fluorescent molecular sensors for determining the effectiveness of the photo-cleavage processes of cationic photoinitiators. Understanding the reactivity of photoinitiators remains a simple way of designing more efficient molecules for the role of cationic photoinitiators, which is a fascinating topic from both an industrial as well as purely scientific point of view.

## Figures and Tables

**Table 1:** Spectral characteristics of the 2,6-diphenylpyridine derivatives.

Acronym	Absorption Characteristics					
	λ_max-ab-UV-C_ [nm]	ε_max_ [dm^3^·mol^−1^·cm^−1^]	λ_max-ab-UV-B_ [nm]	ε_max-UV-B_ [dm^3^·mol^−1^·cm^−1^]	ε_300_ [dm^3^·mol^−1^·cm^−1^]	ε_320_ [dm^3^·mol^−1^·cm^1^]
PT-H	243	19,490	302	8269	8230	3066
PT-OCH_3_	270	21,454	314	10,377	10,802	9913
PT-SCH_3_	292	24,336	317	21,382	24,367	20,962
PT-CH_3_	252	20,381	307	9488	8925	6043
PT-F	244	21,272	303	8417	8297	3528
PT-CN	254	23,302	306	15,590	15,755	11,465
PT-CF_3_	241	27,526	300	12,148	12,148	4262
PT-SO_2_CH_3_	249	27,126	303	12,829	12,743	6805

λ_max-ab-UV-C_ –position of absorption maximum for the short-wavelength band [nm]; ε_max_ –molar extinction coefficient measured at λ_max-ab-UV-C_ [dm^3^·mol^−1^·cm^−1^]; λ_max-ab-UV-B_ –position of absorption maximum for the longer-wavelength band [nm]; ε_max-UV-B_ –molar extinction coefficient measured at λ_max-ab-UV-B_ [dm^3^·mol^−1^·cm^−1^]; ε_300_ –molar extinction coefficient measured at 300 nm [dm^3^·mol^−1^·cm^−1^]; ε_320_–molar extinction coefficient measured at 320 nm [dm^3^·mol^−1^·cm^−1^].

**Table 2:** Spectral characteristics of the 2,6-diphenylpyridine derivatives in acetonitrile.

Acronym	Excitation		Emission at λ = 290 nm **	Δν Stokes Shift [cm^−1^]
	at * [nm]	λ_max-ex_ [nm]	λ_max-em_ [nm]	
PT-H	350	313	341	2623
PT-OCH_3_	360	328	359	2633
PT-SCH_3_	370	340	374	2674
PT-CH_3_	350	318	346	2545
PT-F	350	312	342	2812
PT-CN	350	325	349	2116
PT-CF_3_	350	311	340	2743
PT-SO_2_CH_3_	350	313	345	2963

λ_max-ex_—wavelength for the maximum intensity of the excitation spectrum; λ_max-em_—wavelengths for the maximum intensity of the emission spectrum; * wavelength used to obtain excitation spectra; ** 290 nm is the excitation wavelength, used to obtain emission spectra.

**Table 3:** Summarised data on excitation and emission spectra of PT-SCH_3_ and PT-CN compounds.

Solvent	$E_{N}^{T}$	PT-SCH_3_		PT-CN	
		Excitation Spectrum λ_max-ex_ [nm]	Emission Spectrum λ_max-Fl_ [nm]	Excitation Spectrum λ_max-ex_ [nm]	Emission Spectrum λ_max-Fl_ [nm]
methanol	0.792	339	387	324	347
DMF (dimethylformamide)	0.386	342	372	327	352
DCM (dichloromethane)	0.309	341	369	324	349
chloroform	0.259	339	369	322	349
ethyl acetate	0.228	340	362	323	348
1,4-dioxane	0.164	341	361	324	349

$E_{N}^{T}$ —scale of polarity [51]; λ_max-ex_—wavelength for the maximum intensity of the excitation spectrum [nm]; λ_max-Fl_—wavelengths for the maximum intensity of the emission spectrum [nm].

**Table 4:** Spectroscopic data of 2,6-diphenylpyridine derivatives during the free-radical photopolymerization process.

Sensor	λ_max-BEFORE_ [nm]	Intensity @λ_max-BEFORE_ [a.u.]	λ_max-AFTER POL_ [nm]	Intensity @λ_max-AFTER_ [a.u.]	|ΔI_max_| [a.u.]	ΔI_max_ [%]	Δλ_max_ [nm]
Free-radical photopolymerization process of TMPTA under 320 nm							
PT-H	348	4710	348	6072	1362	29	0
PT-OCH_3_	366	43,331	366	30,018	13,312	31	0
PT-SCH_3_	376	103,347	372	54,747	48,600	47	5
PT-CH_3_	356	21,093	356	18,294	2799	13	0
PT-F	352	9366	352	9394	27	0	0
PT-CN	358	17,255	358	12,291	4964	29	0
PT-CF_3_	340	4026	340	3769	257	6	0
PT-SO_2_CH_3_	364	5191	364	5779	588	11	0

ΔI_max_—changes in fluorescence intensity expressed as a percentage in relation to the initial value before polymerization; |ΔI_max_|—difference in fluorescence intensity before and after the photopolymerization process; Δλ_max_—position of the maximum fluorescence before and after the photopolymerization process.

**Table 5:** Spectroscopic data of the 2,6-diphenylpyridine derivatives during the cationic photopolymerization processes.

Sensor	λ_max-short-BEFORE_ [nm]	I @λ_max-short-BEFORE_ [a.u.]	I @λ_max-short-AFTER_ [a.u.]	λ_max-long_ [nm]	I @λ_max-long-BEFORE_ [a.u.]	I @λ_max-long-AFTER_ [a.u.]	Δλ_max_ [nm]	dI/dt	σ_p_
PT-H	no absorption at λ_max-short_			392	550	13,490	0	179	0.000
PT-OCH_3_	366	24,070	960	440	1330	26,460	74	2034	−0.268
PT-SCH_3_	377	18,180	990	469	590	9380	92	457	0.000
PT-CH_3_	353	2570	1060	406	1080	22,980	53	614	−0.170
PT-F	352	2160	1610	400	670	9880	48	216	0.062
PT-CN	356	1150	2490	441	290	3430	85	12	0.660
PT-CF_3_	no absorption at λ_max-short_			441	270	4000	0	16	0.540
PT-SO_2_CH_3_	no absorption at λ_max-short_			412	260	5490	0	50	0.720

Δλ_max_ = λ_max-long-BEFORE_ - λ_max-short-AFTER_; (dIntensity(_@_λ_max_)/dt)_max_ = dI/dt; σ_p_ - *Hammett substituent constants [54]*.

**Table 6:** Comparison of the maximum intensity and its location for the PT-OCH_3_ compound at different pHs.

pH	PT-OCH_3_			
	λ_max_ [nm]	I_max_ [a.u.]	Δλ_max_ [nm]	ΔI_max_ [a.u.]
H_2_O (distilled water) – reference	364	9.74 × 10^5^	0	0
1	458	1.82 × 10^6^	94	8.44 × 10^5^
2	461	1.70 × 10^6^	97	7.24 × 10^5^
3	354	3.74 × 10^5^	10	1.68 × 10^5^
4	359	5.11 × 10^5^	5	4.63 × 10^5^
5	365	5.94 × 10^5^	1	3.80 × 10^5^
6	367	1.68 × 10^5^	3	8.06 × 10^5^
7	354	3.23 × 10^5^	10	6.51 × 10^5^
8	354	3.67 × 10^5^	10	6.07 × 10^5^
9	360	3.18 × 10^5^	4	6.56 × 10^5^
10	358	2.73 × 10^5^	6	7.00 × 10^5^

λ_max-Fl_—position of maximum fluorescence intensity; I_max_—intensity of the fluorescence at λ_max-Fl_; Δλ_max_—difference between the position of the maximum fluorescence of the PT-OCH_3_ compound in the reference sample (in distilled water) and the maximum of fluorescence in buffers at different pHs [nm]; ΔI_max_—difference between the maximum fluorescence intensity of the PT-OCH_3_ compound in a reference sample (in distilled water) and the maximum intensity of the PT-OCH_3_ compound in buffers at different pHs.

## Supplementary Materials

The following are available online at https://www.mdpi.com/1424-8220/20/11/3043/s1, Figures S1–S16: ^1^H NMR and ^13^C NMR spectra of 2,6-diphenylpyridine derivatives; Figure S17: Synthesized compounds in powder form under: (a) sun light; (b) UV 254 nm light.; Figure S18: Synthesized compounds dissolved in acetonitrile under: (a) sun light; (b) UV 365 nm light; Figures S19–S24: Photolysis of 2,6-diphenylpyridine derivatives in ACN under 300nm (26mW/cm^2^); Figures S25–S31: Photolysis of 2,6-diphenylpyridine derivatives in ACN under 320nm (1mW/cm^2^); Figures S32–S38: Changes of fluorescence spectra of the 2,6-diphenylpyridine derivatives during free-radical photopolymerization of TMPTA monomer under irradiation 320 nm; Figures S39–S40: Photolysis of HIP/PT-OCH_3_ in molar ratio 2:1 and 5.4:1 in ACN upon exposure to LED 320 nm (1mW/cm^2^).
