# Supplementary material for: Double Role of Diphenylpyridine Derivatives as Fluorescent Sensors for Monitoring Photopolymerization and the Determination of the Efficiencies of the Generation of Superacids by Cationic Photoinitiators
Source: Sensors (Basel). 2020 May 27;20(11):3043. doi: 10.3390/s20113043 (PMC7308876; doi:10.3390/s20113043)
Supplement: Supplementary file 1 [file sensors-20-03043-s001.pdf]

Double Role of Diphenylpyridine Derivatives as Fluorescent Sensors for Monitoring Photopolymerization and the Determination of the Efficiencies of the Generation of Superacids by Cationic Photoinitiators

Monika Topa [1], Filip Petko [2], Mariusz Galek [2], and Joanna Ortyl [1,2,*]

[1] Faculty of Chemical Engineering and Technology, Cracow University of Technology, Warszawska 24, 30-155 Cracow, Poland;
[2] Photo HiTech Ltd., Bobrzyńskiego 14, 30-348 Cracow, Poland;

* Correspondence: jortyl@pk.edu.pl (J.O.)

Synthesis - Chemicals and general synthetic procedures of 2,6-diphenylpyridine derivatives

All inorganic salts organic reagents, and solvents were analytically pure and used as received. Structure and purity of obtained products were confirmed by NMR and LC–MS analysis.
^{1}H NMR and ^{13}C NMR spectra were recorded in DMSO–D_6 on Avance III HD 400 MHz (Bruker) spectrometer. Chemical shifts were reported in parts per million (δ) and referenced to residual protonated solvent peak (δ=2.50 ppm in ^{1}H NMR spectra and 39.52 ppm ^{13}C NMR spectra).
LC–MS analyses were obtained on LCMS–2020 (Shimadzu) with ESI ionization method.

Synthesis of 2,6-diphenylpyridine derivatives

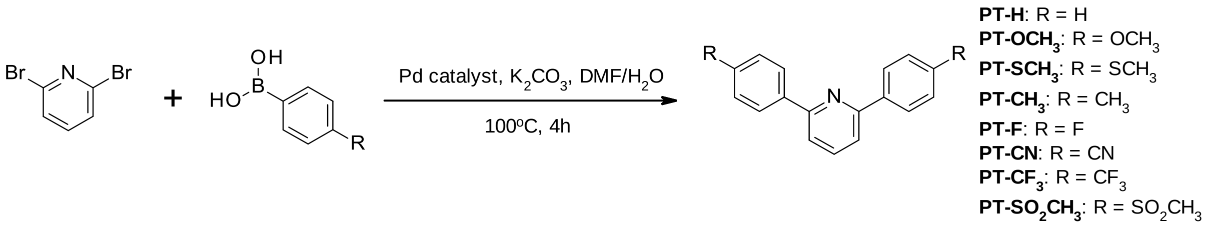

Example: PT-H

2,6-Dibromopyridine (0.85 mmol, 200 mg), Phenylboronic (2.13 mmol, 260 mg), Tetrakis(triphenylphosphine) Palladium (10 %mol, 98 mg) and K_2CO_3 (2.98 mmol, 412 mg) were dissolved in DMF (3.0 cm^3) and H_2O (0.3 cm^3) in pressure tube. Oxygen was removed with argon and mixture was heated for 4 hours in temperature 100°C. When reaction was finished, H_2O was added and product was extracted with EtOAc. Organic phase was washed with brine, dried over Na_2SO_4 and concentrated. Product was purified using flash chromatography followed by recrystallization from methanol.

2,6-diphenylpyridine, ***PT-H*** 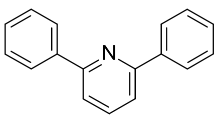	Yield: 0.067 g (34%); ^{1}H NMR (400 MHz, DMSO) δ 8.23 (d, 4H), 8.01 – 7.91 (m, 3H), 7.54 (t, 4H), 7.47 (t, 2H). ^{13}C NMR (101 MHz, DMSO) δ 156.09, 139.14, 138.83, 129.63, 129.25, 127.08, 119.32. MS (ESI) m/z(%): 232.14 (100%, $[M+H]^+$); purity (LC): 99.4%.
2,6-bis(4-methoxyphenyl)pyridine, ***PT-OCH₃*** 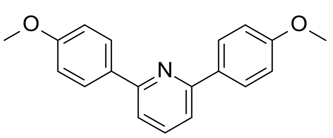	Yield: 0.105 g (42%); ^{1}H NMR (400 MHz, DMSO) δ 8.16 (d, J = 8.5 Hz, 4H), 7.91 – 7.77 (m, 3H), 7.08 (d, J = 8.5 Hz, 4H), 3.84 (s, 6H). ^{13}C NMR (101 MHz, DMSO) δ 160.63, 155.66, 138.50, 131.76, 128.38, 117.65, 114.58, 55.70. MS (ESI) m/z(%): 292.21 (100%, $[M+H]^+$); purity (LC): 100.0%.

2,6-bis(4-methylsulphanylphenyl)pyridine, ***PT-SCH₃*** 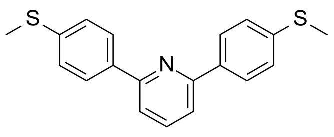	Yield: 0.160 g (58%);<br>^{1}H NMR (400 MHz, DMSO) δ 8.17 (d, J = 8.5 Hz, 4H), 7.97 – 7.87 (m, 3H), 7.41 (d, J = 8.5 Hz, 4H), 2.55 (s, 6H).<br>^{13}C NMR (101 MHz, DMSO) δ 155.49, 140.19, 138.77, 135.56, 127.44, 126.31, 118.64, 14.90.<br>MS (ESI) m/z(%): 324.18 (100%, $[M+H]^{+}$); purity (LC): 99.6%.
2,6-bis(p-tolyl)pyridine, ***PT-CH₃*** 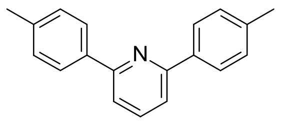	Yield: 0.139 g (63%);<br>^{1}H NMR (400 MHz, DMSO) δ 8.11 (d, *J* = 8.1 Hz, 4H), 7.91 (m, 6.6 Hz, 3H), 7.34 (d, *J* = 8.0 Hz, 4H), 2.39 (s, 6H).<br>^{13}C NMR (101 MHz, DMSO) δ 155.97, 139.09, 138.63, 136.46, 129.83, 126.94, 118.61, 21.34.<br>MS (ESI) m/z(%): 260.18 (100%, $[M+H]^{+}$); purity (LC): 100.0%.
2,6-bis(4-fluorophenyl)pyridine, ***PT-F*** 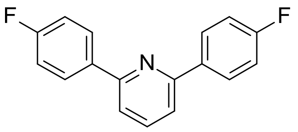	Yield: 0.059 g (26%);<br>1H NMR (400 MHz, DMSO) δ 8.17 (d, J = 8.5 Hz, 4H), 7.97 – 7.87 (m, 3H), 7.41 (d, J = 8.5 Hz, 4H), 2.55 (s, 6H).<br>^{13}C NMR (101 MHz, DMSO) δ 164.64, 162.20, 155.06, 139.00, 135.53, 129.28, 119.09, 116.21, 116.00.<br>MS (ESI) m/z(%): 268.16 (100%, $[M+H]^{+}$); purity (LC): 100.0%.
2,6-bis(4-cyanophenyl)pyridine, ***PT-CN*** 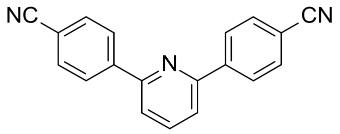	Yield: 0.151 g (63%);<br>^{1}H NMR (400 MHz, DMSO) δ 8.45 (d, *J* = 8.1 Hz, 4H), 8.17 – 8.11 (m, 3H), 7.91 (d, *J* = 8.2 Hz, 4H).<br>^{13}C NMR (101 MHz, DMSO) δ 154.43, 142.90, 139.54, 133.29, 127.92, 121.43, 119.24, 112.22.<br>MS (ESI) m/z(%): 282.18 (100%, $[M+H]^{+}$); purity (LC): 98.3%.
2,6-bis[4-(trifluoromethyl)phenyl]pyridine, ***PT-CF₃*** 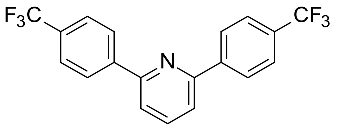	Yield: 0.192 g (61%);<br>^{1}H NMR (400 MHz, DMSO) δ 8.31 – 8.24 (m, 4H), 8.01 – 7.90 (m, 3H), 7.36 (t, *J* = 8.9 Hz, 4H).<br>^{13}C NMR (101 MHz, DMSO) δ 154.82, 142.68, 139.48, 130.04, 129.72, 127.93, 126.18, 123.39, 121.09.<br>MS (ESI) m/z(%): 368.18 (100%, $[M+H]^{+}$); purity (LC): 95.3%.
2,6-bis(4-methylsulphonylphenyl)pyridine, ***PT-SO₂CH₃*** 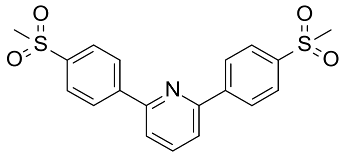	Yield: 0.105 g (32%);<br>^{1}H NMR (400 MHz, DMSO) δ 8.49 (d, *J* = 8.4 Hz, 4H), 8.22 – 7.99 (m, 7H), 3.30 (s, 6H).<br>^{13}C NMR (101 MHz, DMSO) δ 154.80, 143.49, 141.67, 139.55, 128.69, 128.18, 128.05, 121.50, 44.00.<br>MS (ESI) m/z(%): 388.12 (100%, $[M+H]^{+}$); purity (LC): 93.8%.

NMR spectra of synthesized 2,6-diphenylpyridine

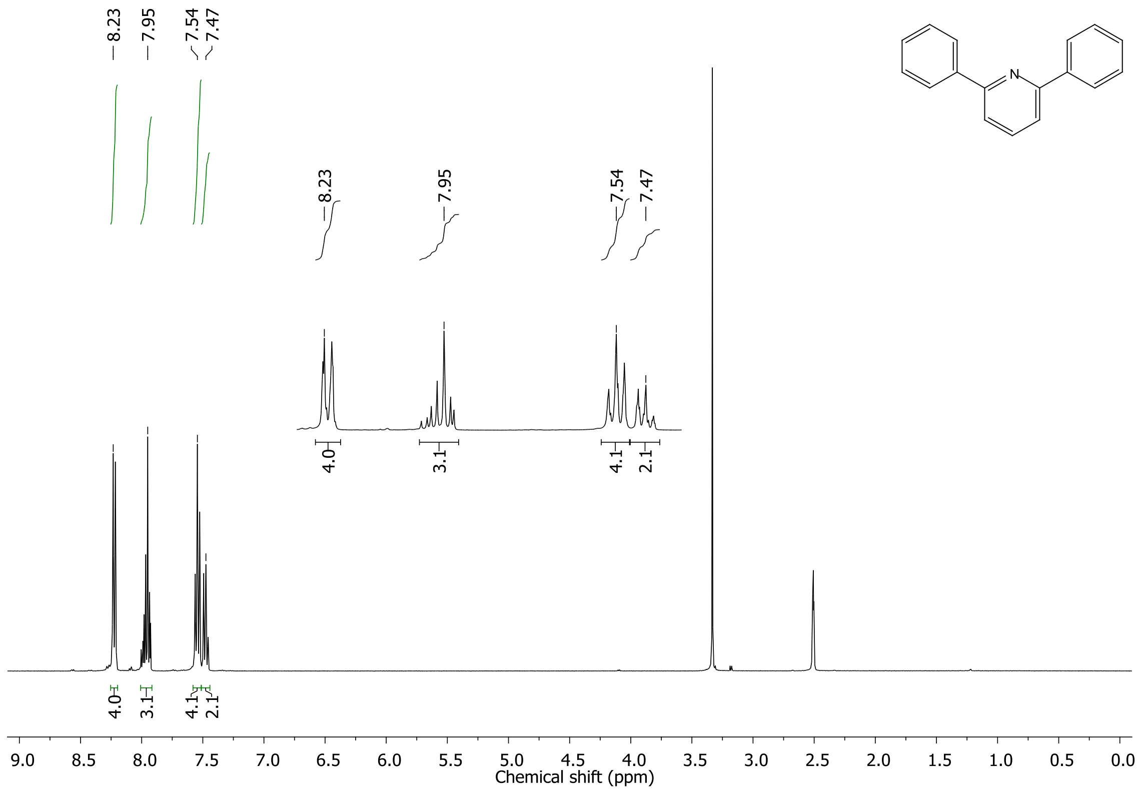

Figure S1: ^{1}H NMR spectrum of 2,6-diphenylpyridine, ***PT-H***.

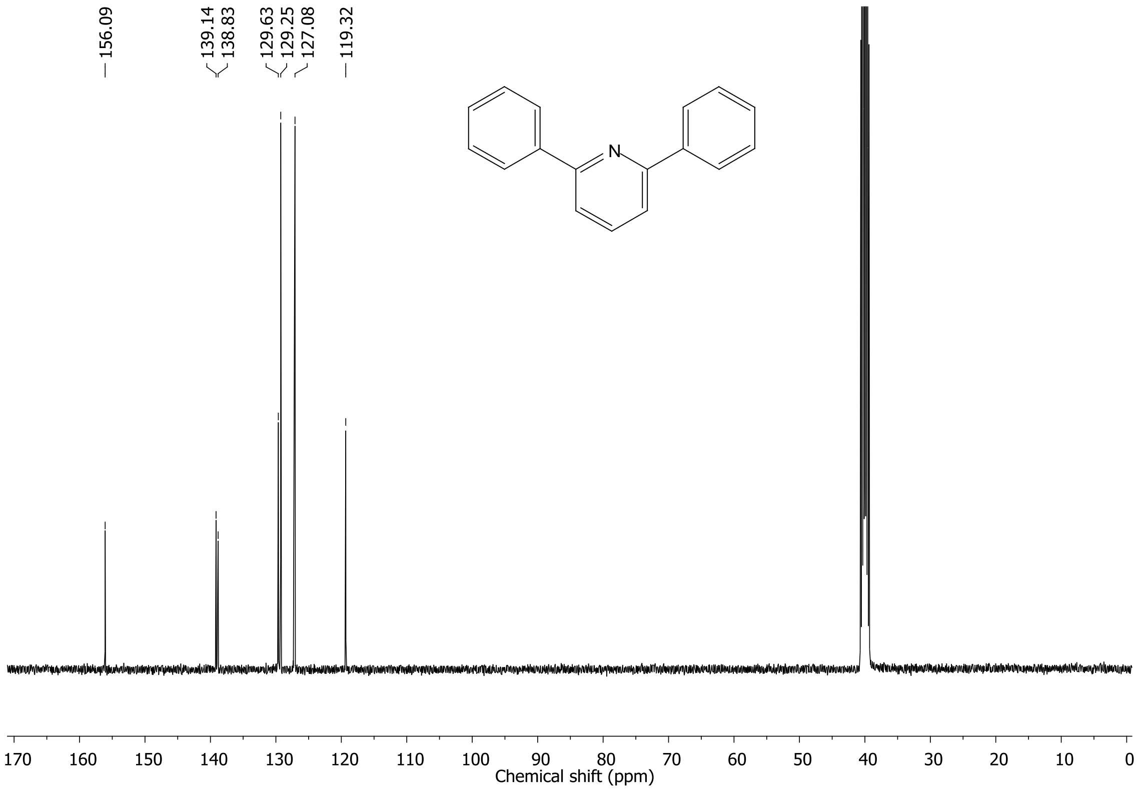

Figure S2: ^{13}C NMR spectrum of 2,6-diphenylpyridine, ***PT-H***.

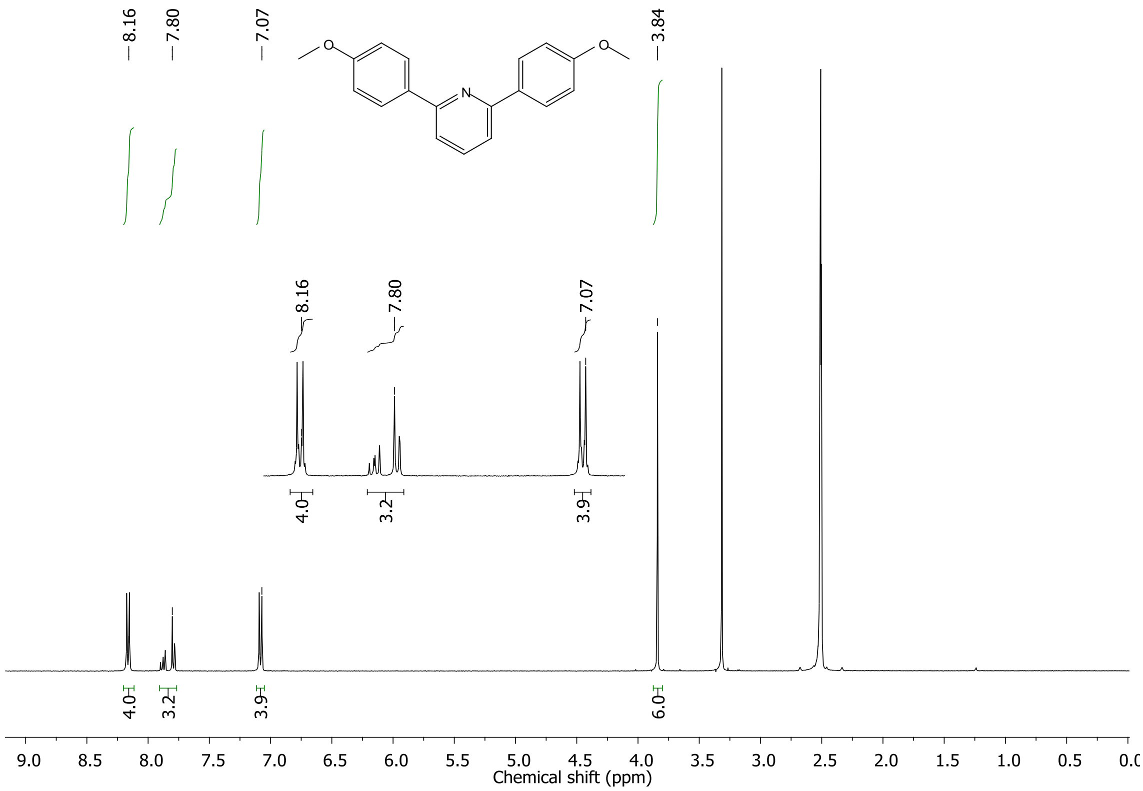

Figure S3: ^{1}H NMR spectrum of 2,6-bis(4-methoxyphenyl)pyridine ***PT-OCH₃***.

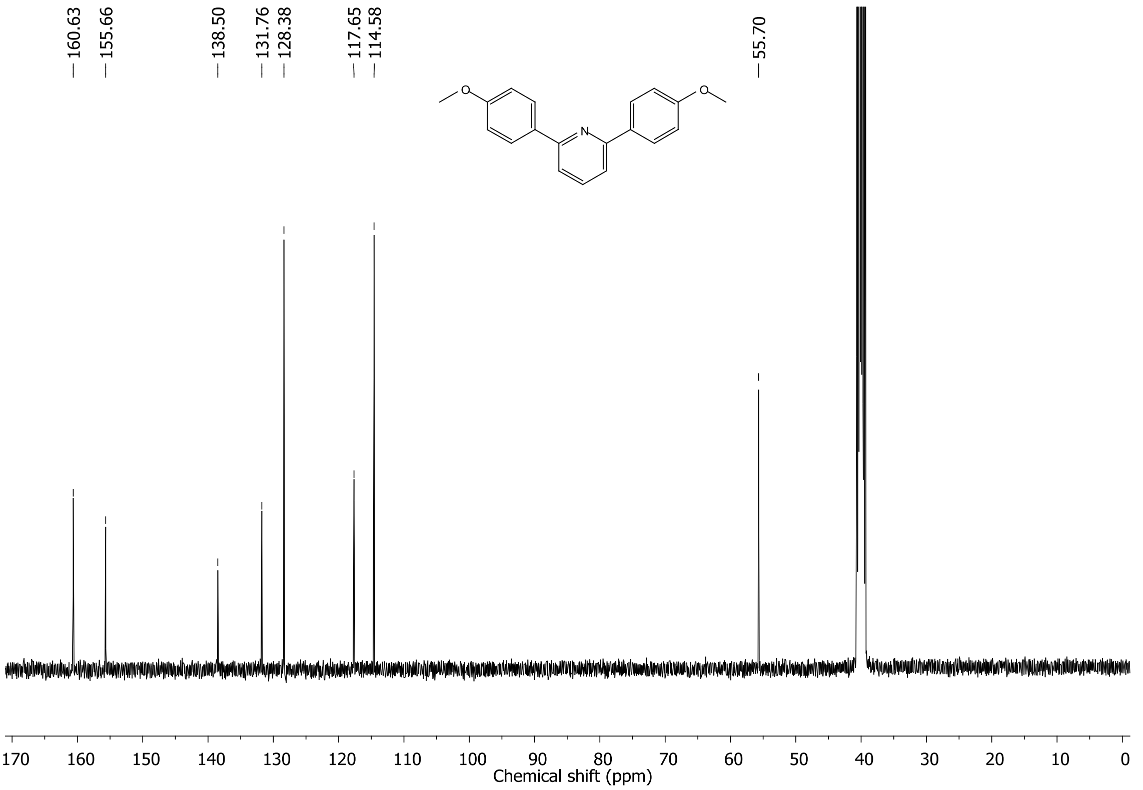

Figure S4: ^{13}C NMR spectrum of 2,6-bis(4-methoxyphenyl)pyridine ***PT-OCH₃***.

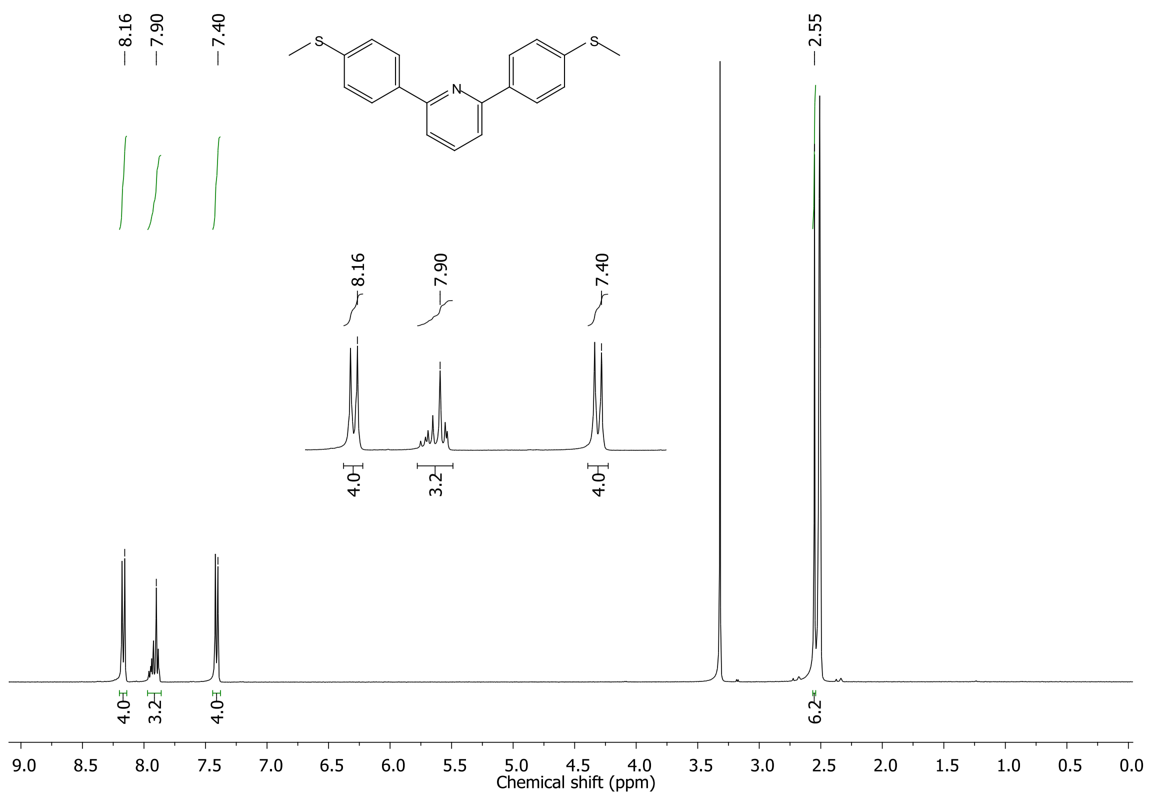

Figure S5: ^{1}H NMR spectrum of 2,6-bis(4-methylsulphanylphenyl)pyridine ***PT-SCH₃***.

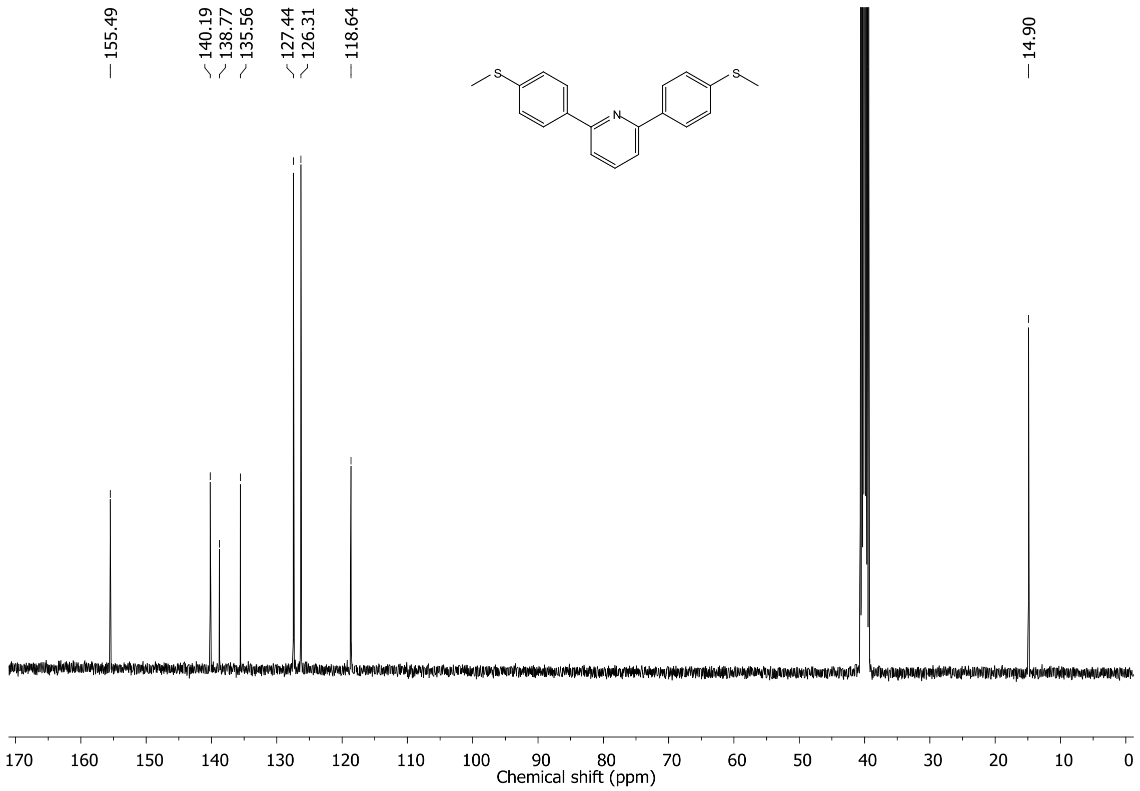

Figure S6: ^{13}C NMR spectrum of 2,6-bis(4-methylsulphanylphenyl)pyridine ***PT-SCH₃***.

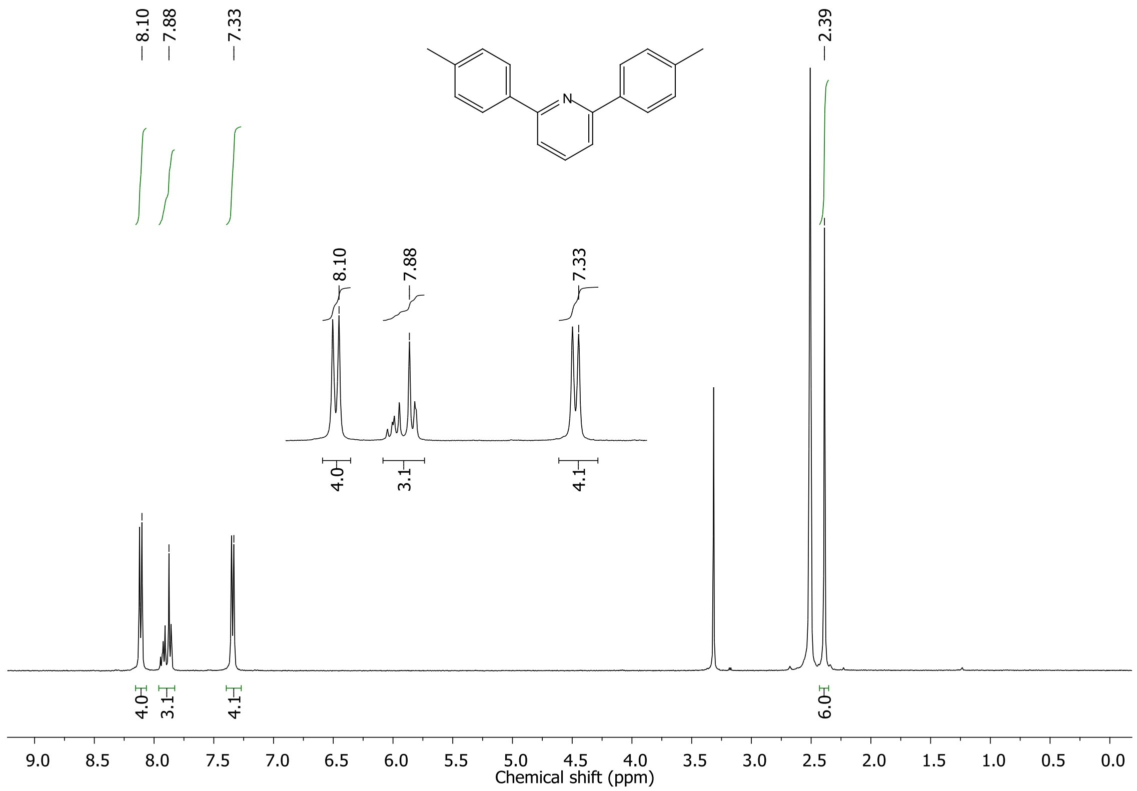

Figure S7: ^{1}H NMR spectrum of 2,6-bis(p-tolyl)pyridine ***PT-CH₃***.

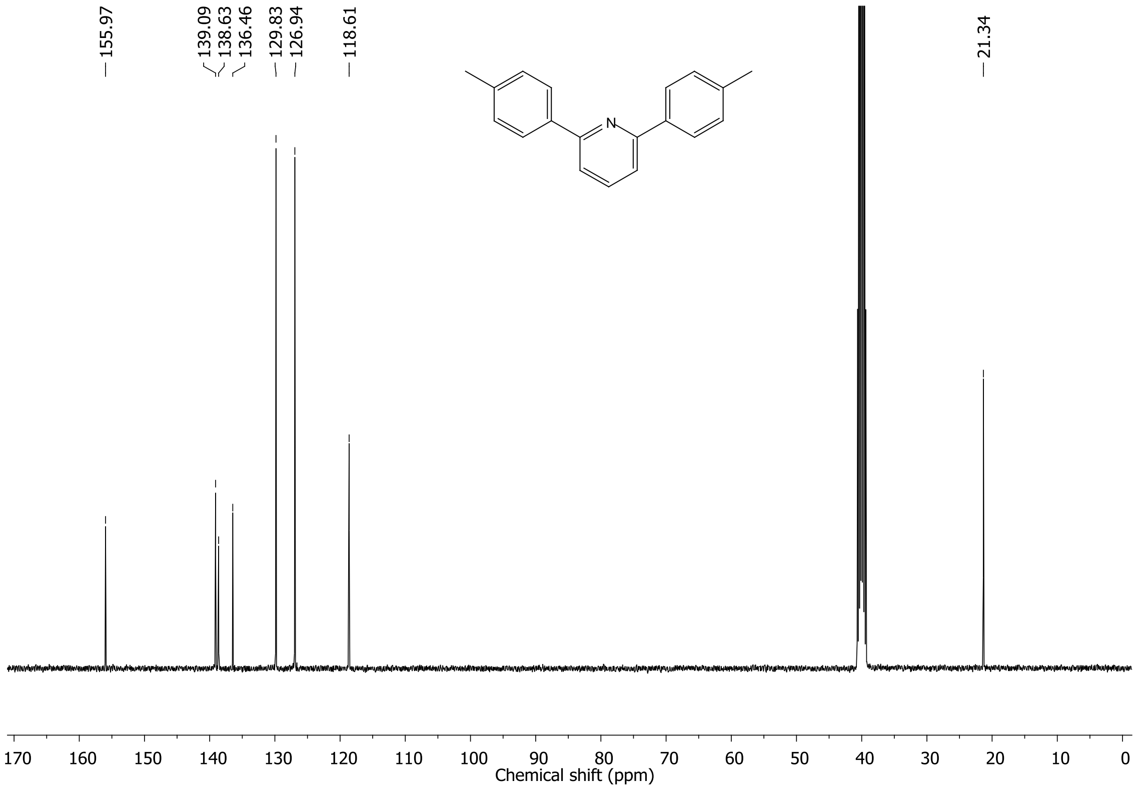

Figure S8: ^{13}C NMR spectrum of 2,6-bis(p-tolyl)pyridine ***PT-CH₃***.

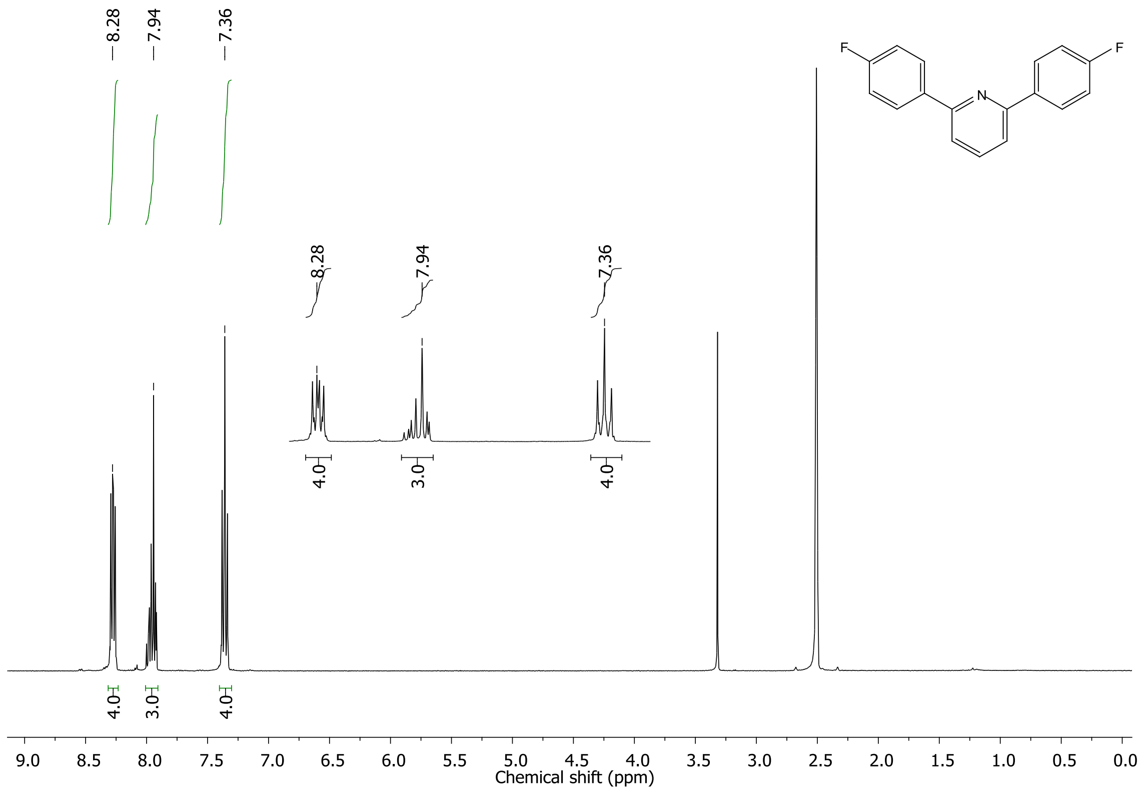

Figure S9: ^{1}H NMR spectrum of 2,6-bis(4-fluorophenyl)pyridine ***PT-F***.

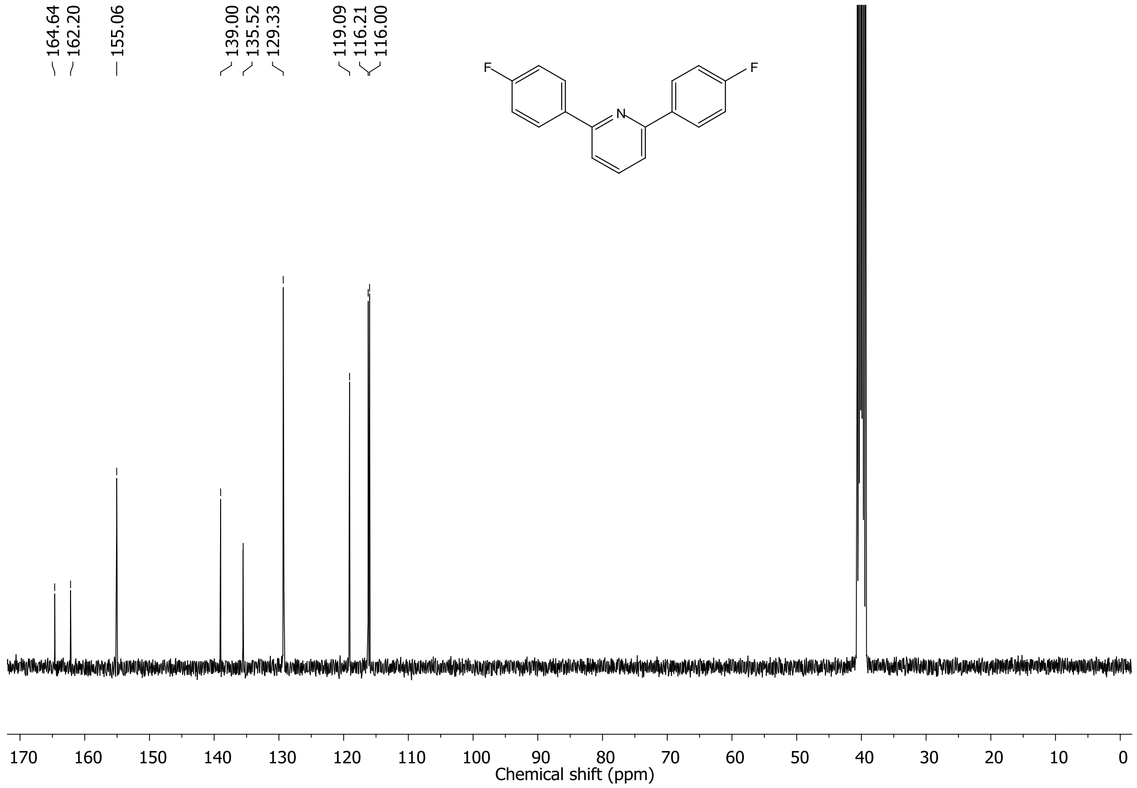

Figure S10: ^{13}C NMR spectrum of 2,6-bis(4-fluorophenyl)pyridine ***PT-F***.

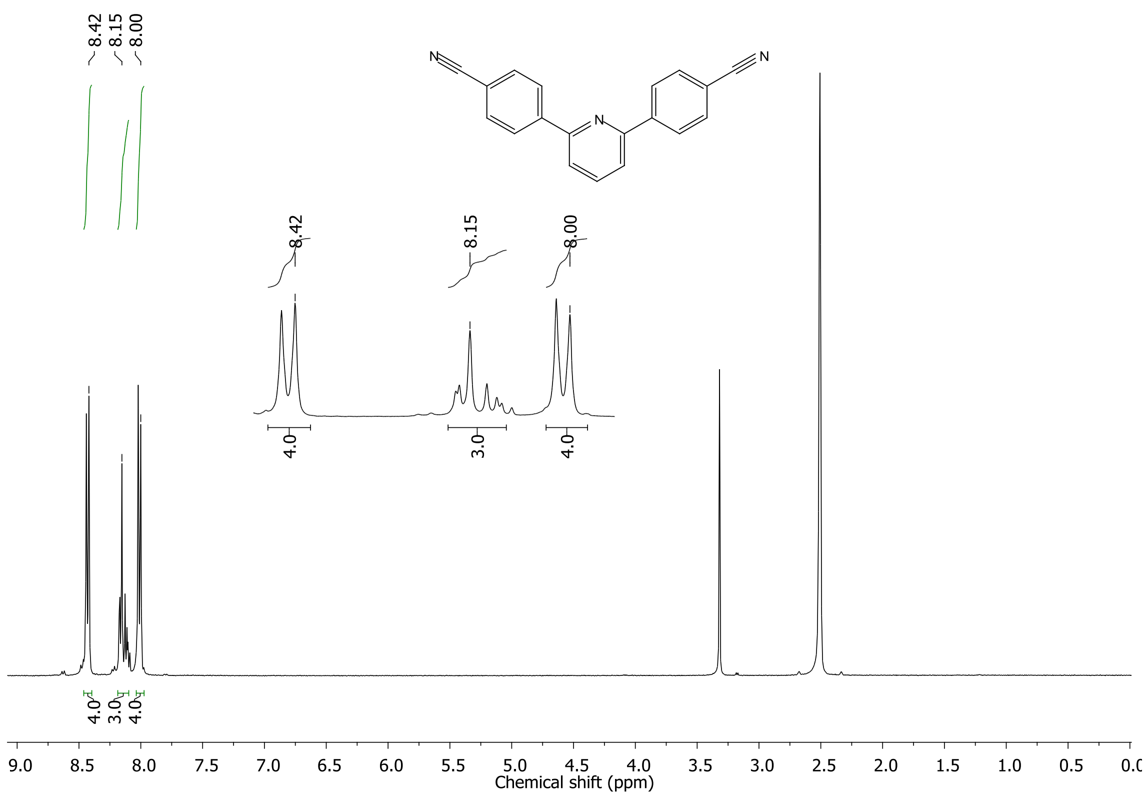

Figure S11: ^{1}H NMR spectrum of 2,6-bis(4-cyanophenyl)pyridine ***PT-CN***.

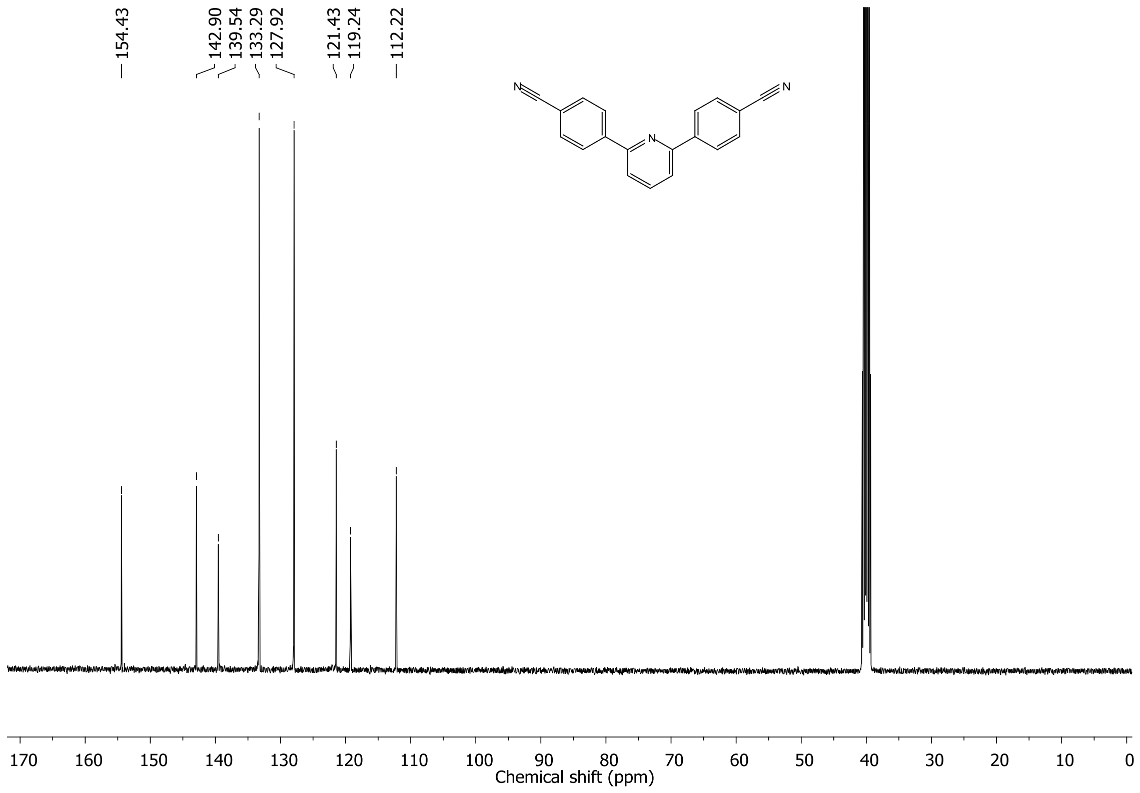

Figure S12: ^{13}C NMR spectrum of 2,6-bis(4-cyanophenyl)pyridine ***PT-CN***.

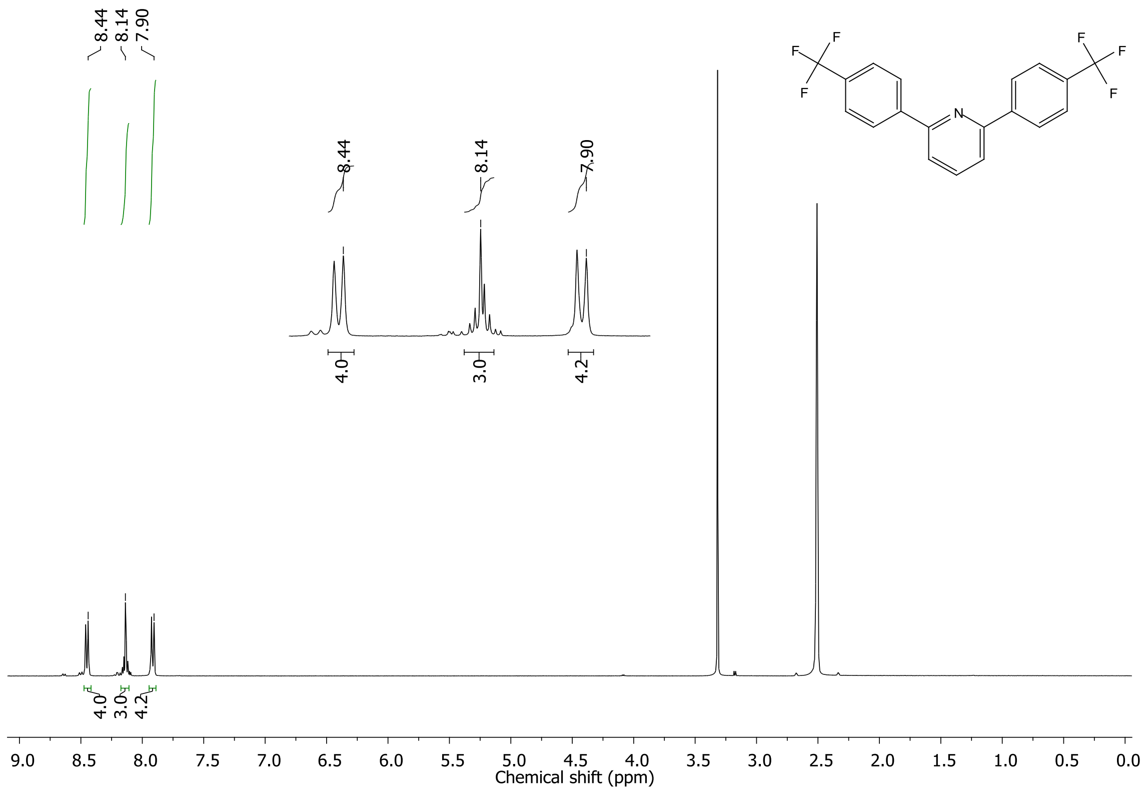

Figure S13: ^{1}H NMR spectrum of 2,6-bis[4-(trifluoromethyl)phenyl]pyridine ***PT-CF*$_3$**.

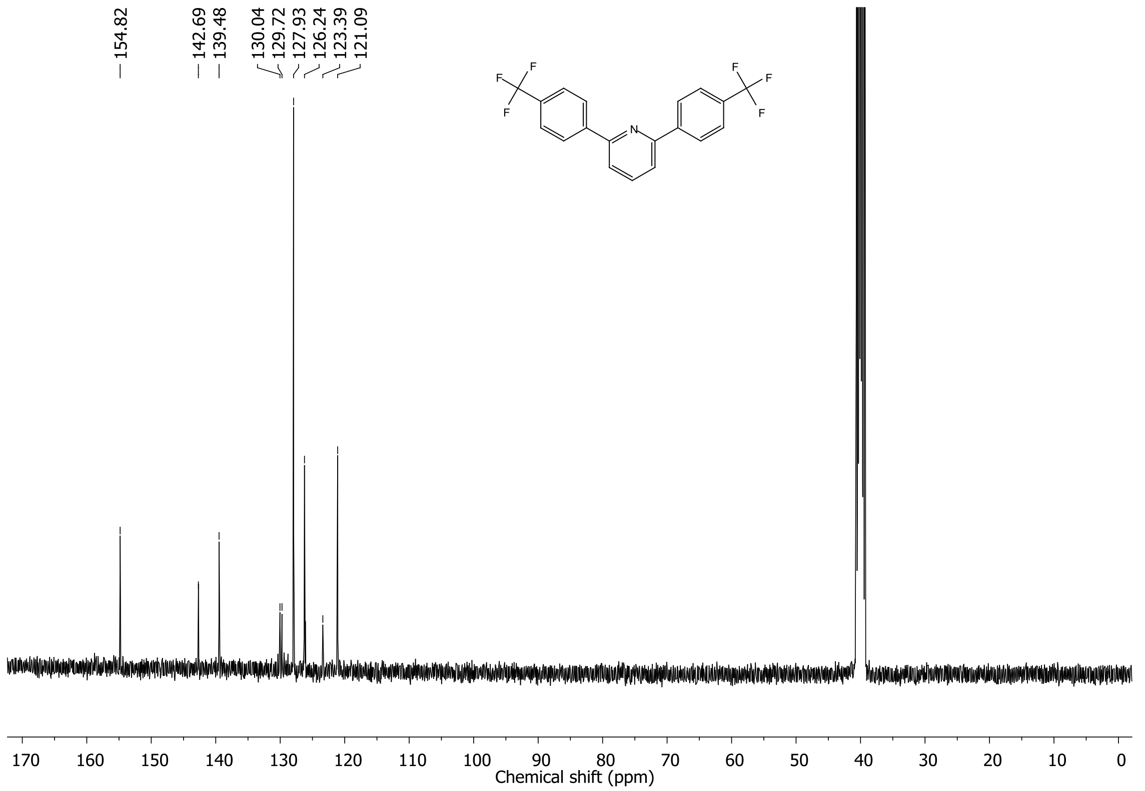

Figure S14: ^{13}C NMR spectrum of 2,6-bis[4-(trifluoromethyl)phenyl]pyridine ***PT-CF*$_3$**.

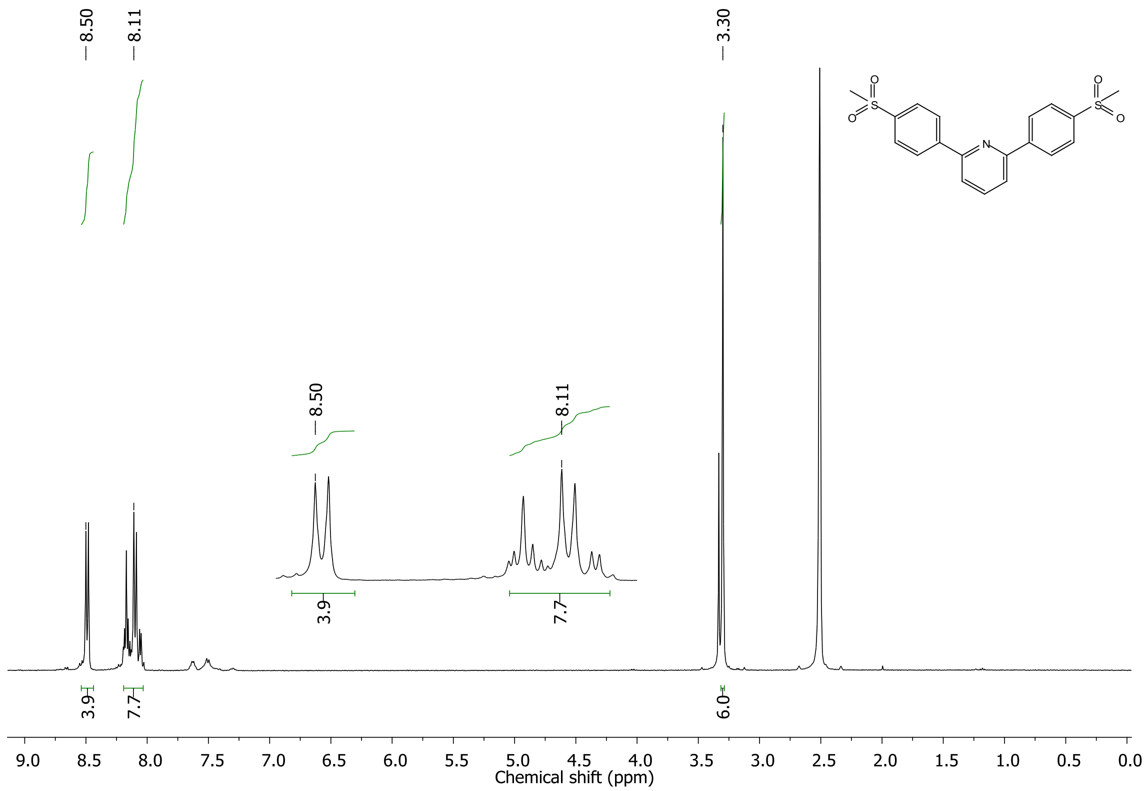

Figure S15: ^{1}H NMR spectrum of 2,6-bis(4-methylsulphonylphenyl)pyridine ***PT-SO$_2$CH$_3$***.

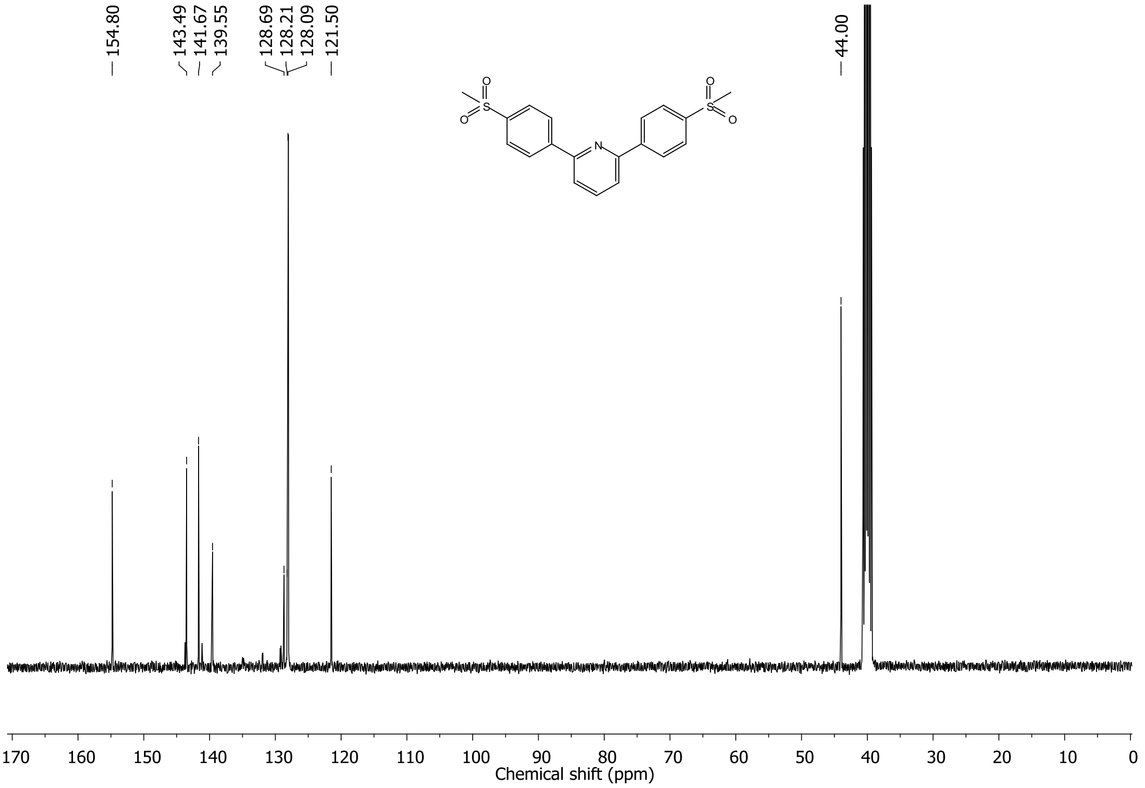

Figure S16: ^{13}C NMR spectrum of 2,6-bis(4-methylsulphonylphenyl)pyridine ***PT-SO$_2$CH$_3$***.

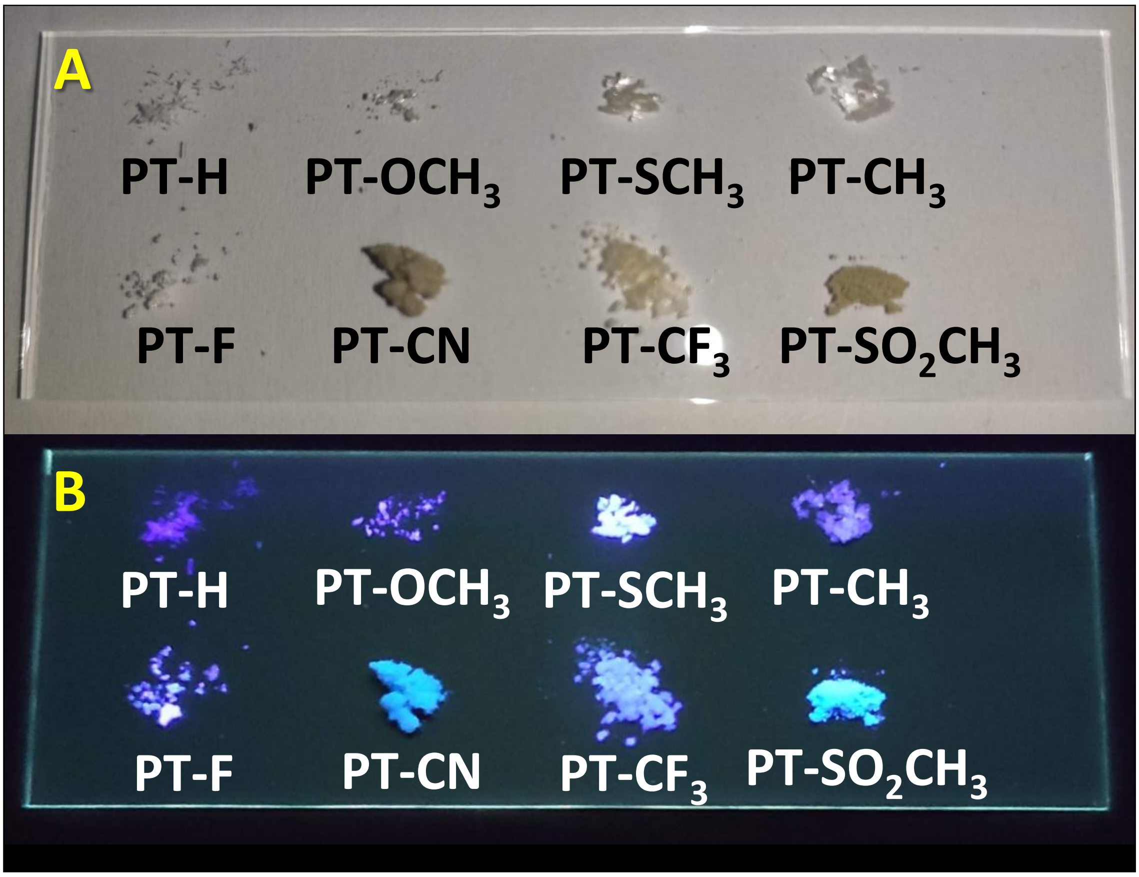

Figure S17: Synthesized compounds in powder form under: (**a**) sun light; (**b**) UV 254 nm light.

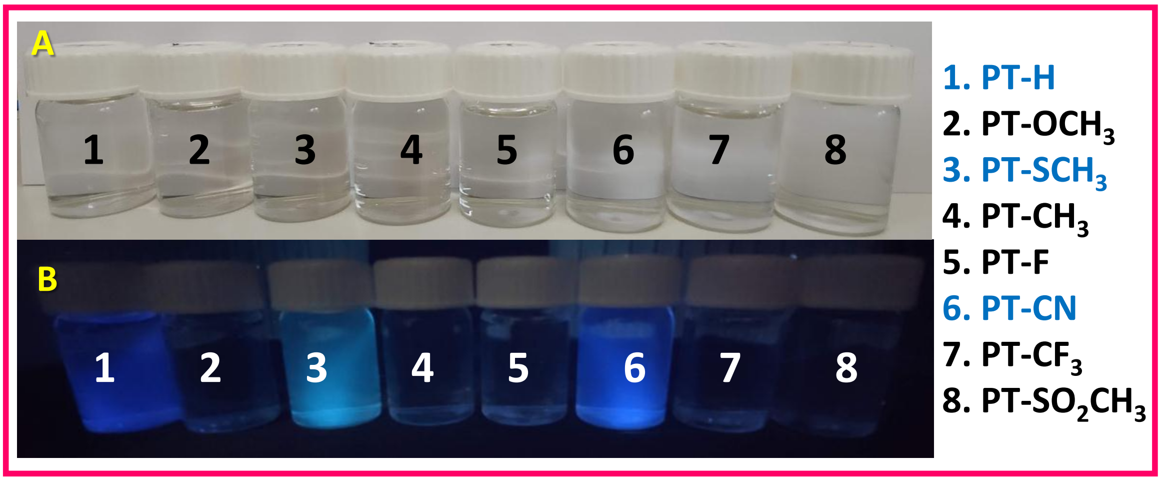

Figure S18: Synthesized compounds dissolved in acetonitrile under: (**a**) sun light; (**b**) UV 365 nm light.

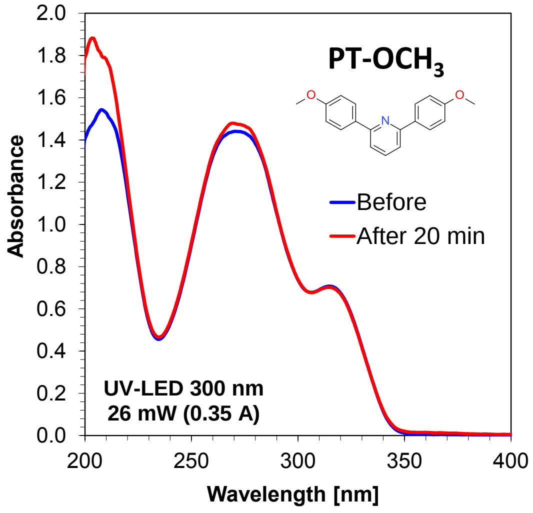

Figure S19: Photolysis of PT-OCH$_3$ (concentration: 6.87·10^{-5} [mol/dm^3]) in ACN under 300nm (26mW/cm^2).

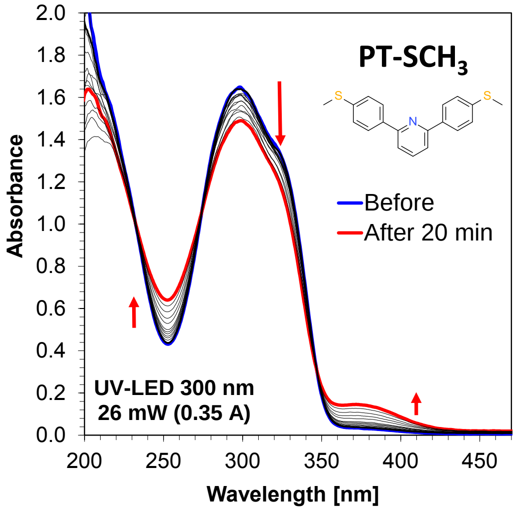

Figure S20: Photolysis of PT-SCH$_3$ (concentration: 6.38·10^{-5} [mol/dm^3]) in ACN under 300nm (26mW/cm^2).

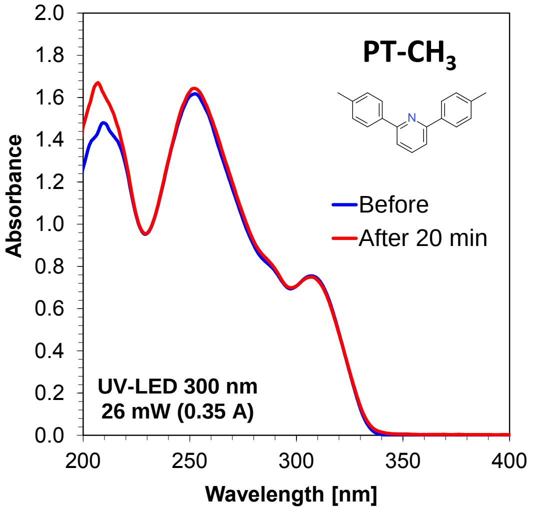

Figure S21: Photolysis of PT-CH$_3$ (concentration: 8.03·10^{-5} [mol/dm^3]) in ACN under 300nm (26mW/cm^2).

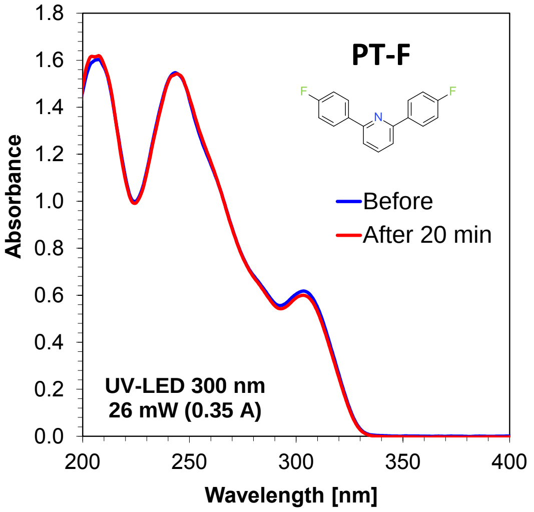

Figure S22: Photolysis of PT-F (concentration: 7.34·10^{-5} [mol/dm^3]) in ACN under 300nm (26mW/cm^2).

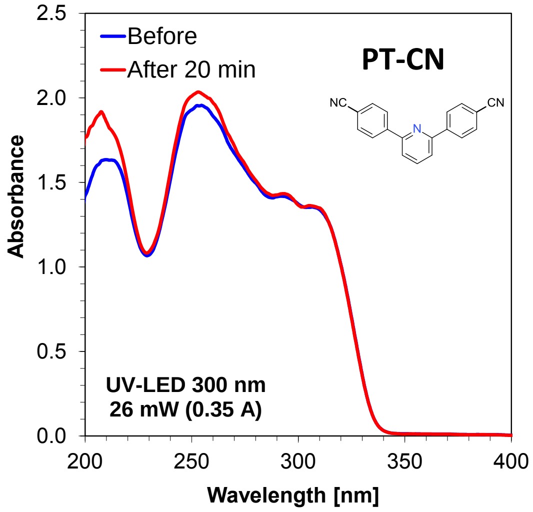

Figure S23: Photolysis of PT-CN (concentration: 8.61·10^{-5} [mol/dm^3]) in ACN under 300nm (26mW/cm^2).

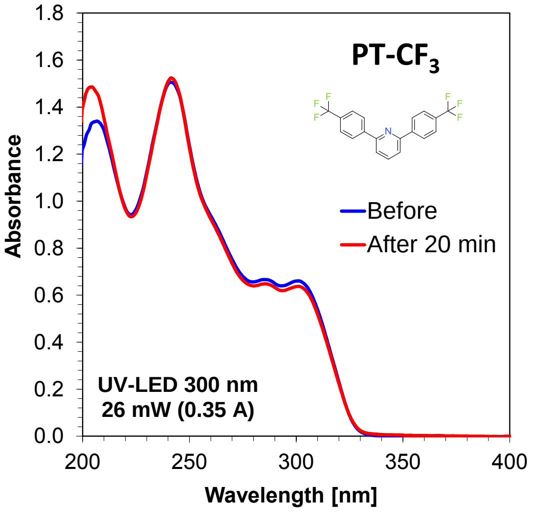

Figure S24: Photolysis of PT-CF$_3$ (concentration: 5.56·10^{-5} [mol/dm^3]) in ACN under 300nm (26mW/cm^2).

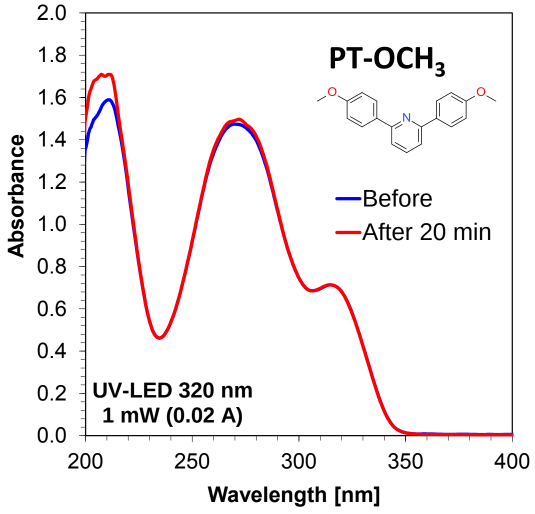

Figure S25: Photolysis of PT-OCH$_3$ (concentration: 6.87·10^{-5} [mol/dm^3]) in ACN under 320nm (1mW/cm^2).

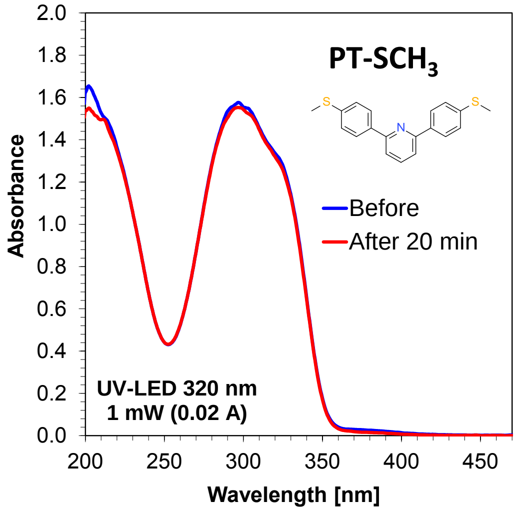

Figure S26: Photolysis of PT-SCH$_3$ (concentration: 6.38·10^{-5} [mol/dm^3]) in ACN under 320nm (1mW/cm^2).

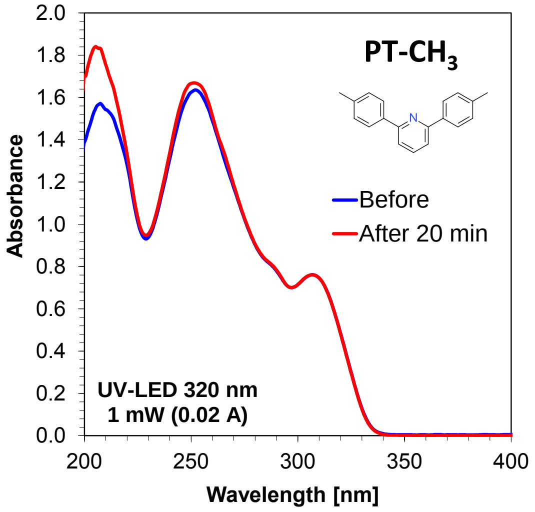

Figure S27: Photolysis of PT-CH$_3$ (concentration: 8.03·10^{-5} [mol/dm^3]) in ACN under 320nm (1mW/cm^2).

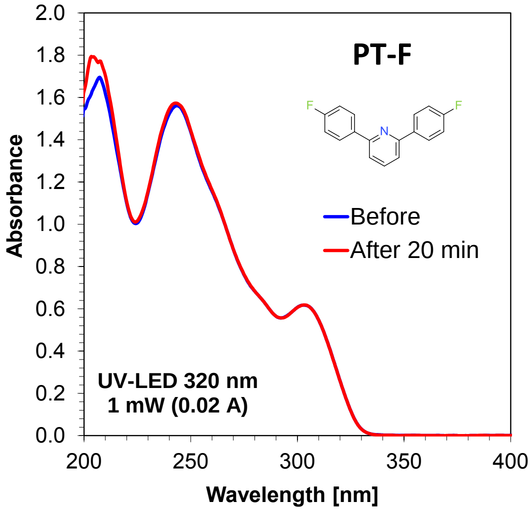

Figure S28: Photolysis of PT-F (concentration: 7.34·10^{-5} [mol/dm^3]) in ACN under 320nm (1mW/cm^2).

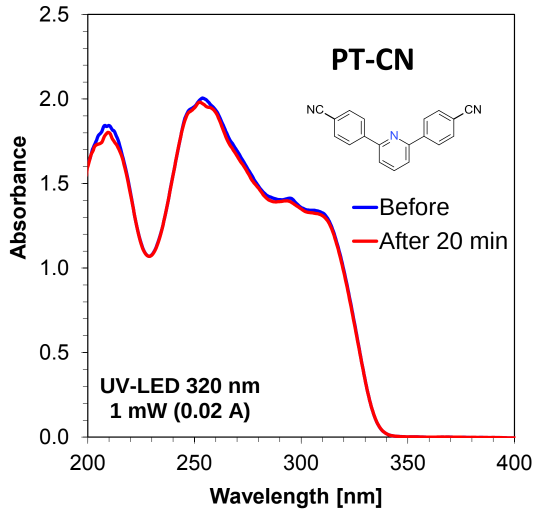

Figure S29: Photolysis of PT-CN (concentration: $8.61 \cdot 10^{-5}$ [mol/dm³]) in ACN under 320nm (1mW/cm²).

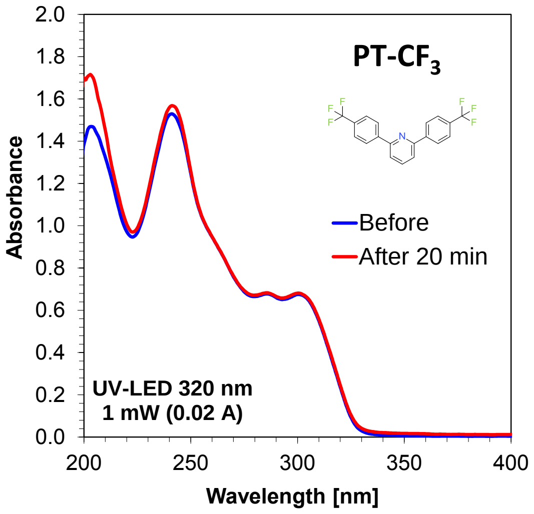

Figure S30: Photolysis of PT-CF₃ (concentration: $5.56 \cdot 10^{-5}$ [mol/dm³]) in ACN under 320nm (1mW/cm²).

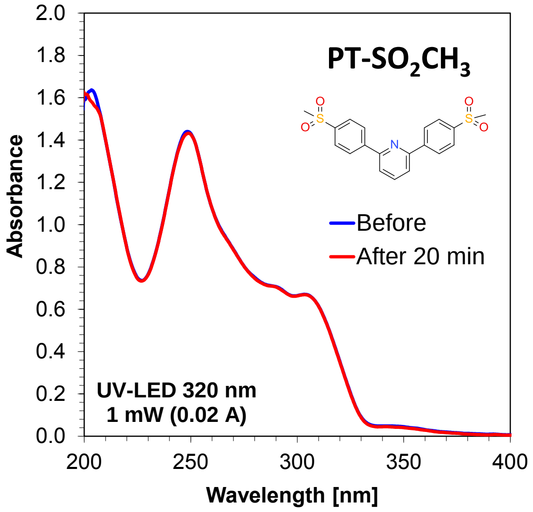

Figure S31: Photolysis of PT-SO_2CH_3 (concentration: $5.17 \cdot 10^{-5}$ [mol/dm³]) in ACN under 320nm (1mW/cm²).

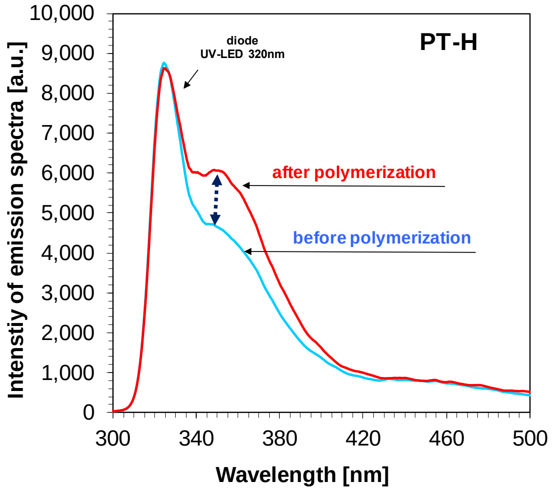

Figure S32: Changes of fluorescence spectra of the PT-H sensor during free-radical photopolymerization of TMPTA monomer under irradiation 320 nm.

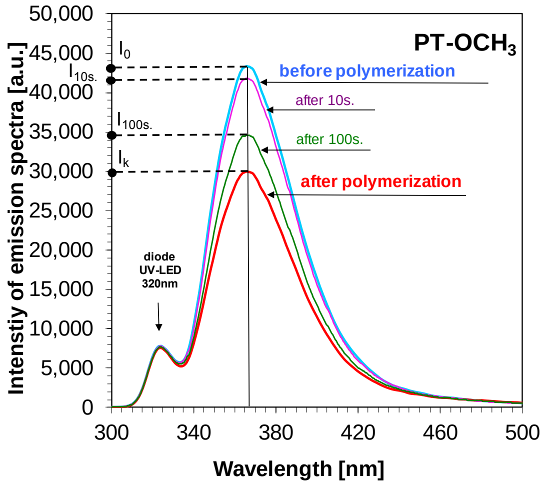

Figure S33: Changes of fluorescence spectra of the PT-OCH3 sensor during free-radical photopolymerization of TMPTA monomer under irradiation 320 nm.

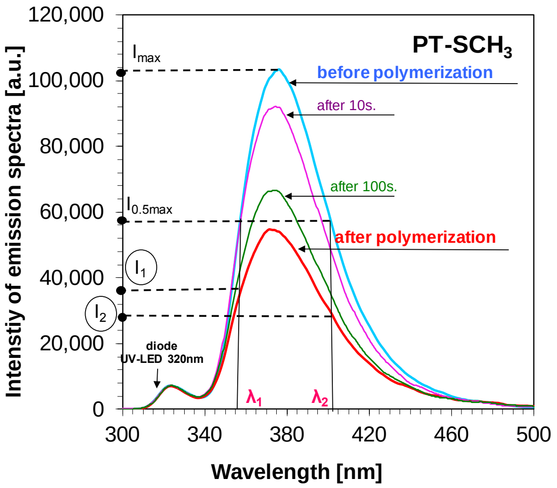

Figure S34: Changes of fluorescence spectra of the PT-SCH3 sensor during free-radical photopolymerization of TMPTA monomer under irradiation 320 nm, (λ_1, λ_2 are monitoring wavelengths).

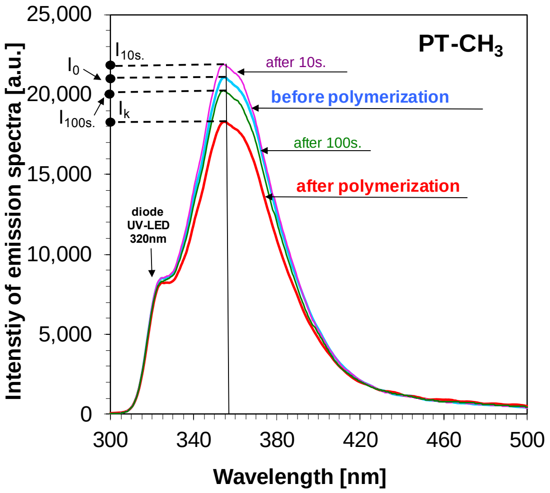

Figure S35: Changes of fluorescence spectra of the PT-CH3 sensor during free-radical photopolymerization of TMPTA monomer under irradiation 320 nm.

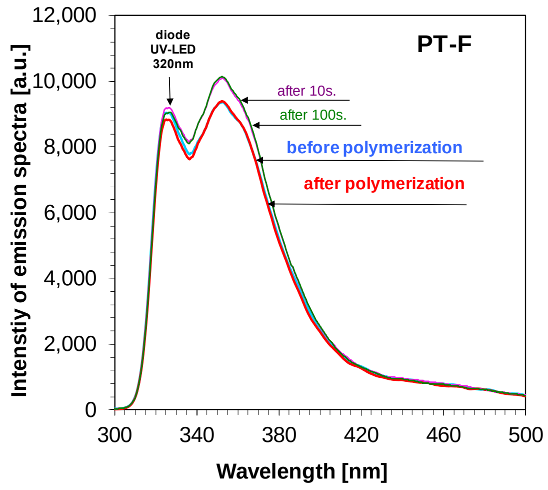

Figure S36: Changes of fluorescence spectra of the PT-F sensor during free-radical photopolymerization of TMPTA monomer under irradiation 320 nm.

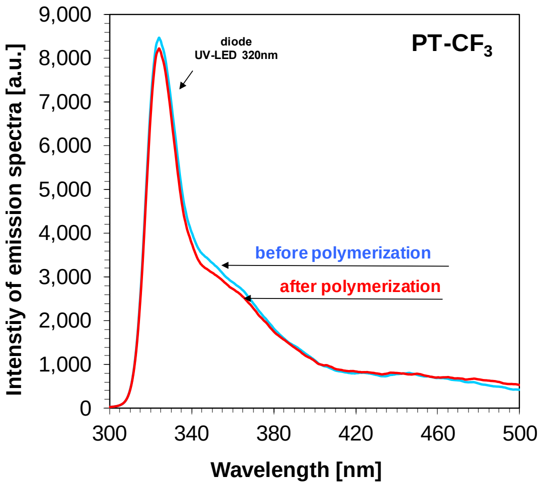

Figure S37: Changes of fluorescence spectra of the PT-CF_3 sensor during free-radical photopolymerization of TMPTA monomer under irradiation 320 nm.

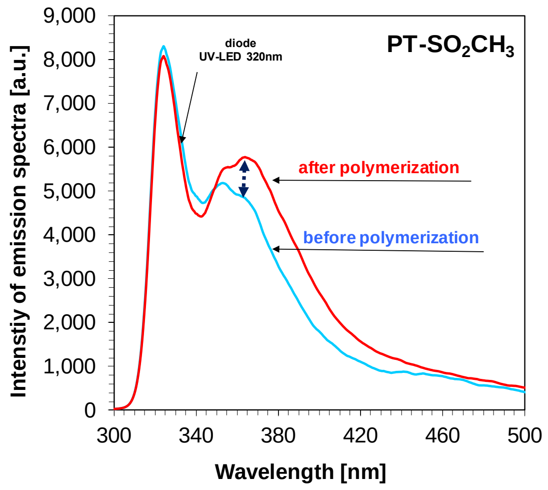

Figure S38: Changes of fluorescence spectra of the PT-SO_2CH_3 sensor during free-radical photopolymerization of TMPTA monomer under irradiation 320 nm.

Photolysis of PT-OCH_3 with different amount of HIP

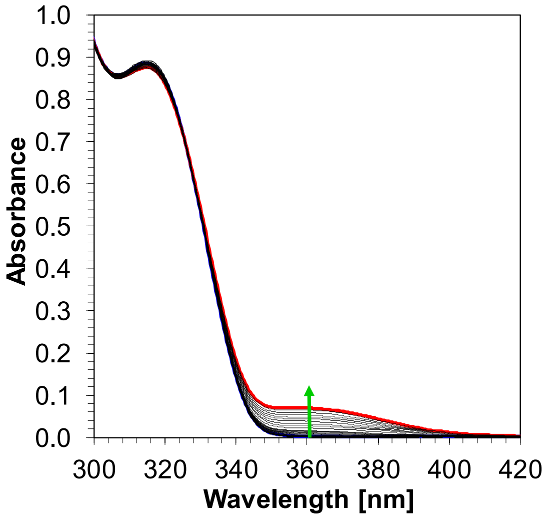

Figure 39: Photolysis of HIP/PT-OCH_3 in molar ratio 2:1 in ACN upon exposure to LED 320 nm ($1mW/cm^2$).

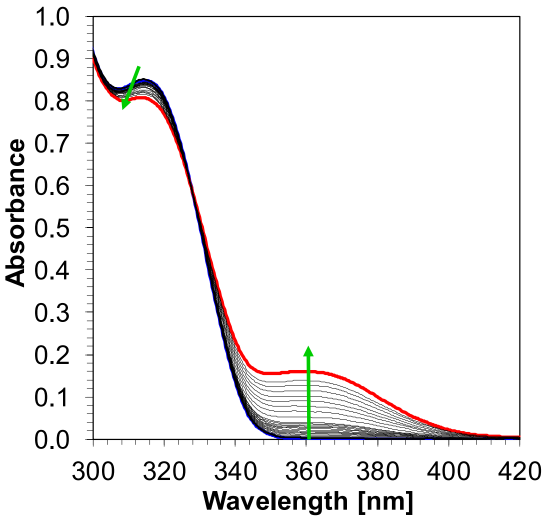

Figure 40: Photolysis of HIP/PT-OCH_3 in molar ratio 5.4:1 in ACN upon exposure to LED 320 nm ($1mW/cm^2$).
